# Cyanotoxins: Bioaccumulation and Effects on Aquatic Animals

**DOI:** 10.3390/md9122729

**Published:** 2011-12-16

**Authors:** Aloysio da S. Ferrão-Filho, Betina Kozlowsky-Suzuki

**Affiliations:** 1 Laboratory of Evaluation and Promotion of Environmental Health, Instituto Oswaldo Cruz, FIOCRUZ, Av. Brasil 4365, Manguinhos, Rio de Janeiro, RJ 21045-900, Brazil; 2 Departament of Ecology and Marine Resources, Federal University of Rio de Janeiro State (UNIRIO), Av. Pasteur 458, Urca, Rio de Janeiro, RJ 22290-040, Brazil; Email: betinaksuzuki@unirio.br

**Keywords:** cyanobacteria, cyanotoxins, bioaccumulation, invertebrates, vertebrates

## Abstract

Cyanobacteria are photosynthetic prokaryotes with wide geographic distribution that can produce secondary metabolites named cyanotoxins. These toxins can be classified into three main types according to their mechanism of action in vertebrates: hepatotoxins, dermatotoxins and neurotoxins. Many studies on the effects of cyanobacteria and their toxins over a wide range of aquatic organisms, including invertebrates and vertebrates, have reported acute effects (e.g., reduction in survivorship, feeding inhibition, paralysis), chronic effects (e.g., reduction in growth and fecundity), biochemical alterations (e.g., activity of phosphatases, GST, AChE, proteases), and behavioral alterations. Research has also focused on the potential for bioaccumulation and transferring of these toxins through the food chain. Although the herbivorous zooplankton is hypothesized as the main target of cyanotoxins, there is not unquestionable evidence of the deleterious effects of cyanobacteria and their toxins on these organisms. Also, the low toxin burden in secondary consumers points towards biodilution of microcystins in the food web as the predominant process. In this broad review we discuss important issues on bioaccumulation and the effects of cyanotoxins, with emphasis on microcystins, as well as drawbacks and future needs in this field of research.

## 1. Introduction

Cyanobacteria or blue-green algae comprise a diverse group of prokaryotic organisms. Their long history of evolution on Earth (~3.5 billions of years) has enabled them to live in different environments and occupy distinct niches. They can be found from the poles to tropical regions, in both terrestrial and marine ecosystems. Several adaptations such as the ability to fix atmospheric-N through in specialized structures called heterocytes, to form resistant cells that work as spores (acinets), to regulate their position in the water column through specialized structures, to tolerate high UV exposure, high metal concentrations, low oxygen levels and a wide range of temperatures [1] allow such broad geographical distribution and the occupation of different niches. However, the most intriguing characteristic of cyanobacteria is their ability to produce secondary metabolites designated as cyanotoxins [2].

Cyanotoxins can be divided into two main criteria: (1) on the basis of their mechanism of action on terrestrial vertebrates, especially mammals—e.g., hepatotoxins, neurotoxins, dermatotoxins, *etc.*; and (2) according to their chemical structure—e.g., cyclic peptides, alkaloids or lipopolyssacharides [2,3,4]. Anatoxin-a—ANTX-a—was the first described cyanotoxin belonging to the class of the neurotoxins [2]. This alkaloid is a potent post-synaptic cholinergic nicotinic agonist and neuromuscular blocking agent [3]. By irreversibly binding to the acetylcholine receptors and not being degraded by acetylcholinesterase, it causes staggering, muscle twitching and gasping in animals, opisthotonus in birds (*i.e.*, head and neck stretched backwards along the back), and rapid death by respiratory arrest [2]. Anatoxin-a(s)—ANTX-a(s)—has a different mode of action. As any organophosphate compound, ANTX-a(s) inhibits the activity of the acetylcholinesterase, causing hypersalivation and convulsions in animals and also death by respiration arrest [4]. Both toxins act very fast, killing mice in a few minutes (2–30 min) after intraperitonial (i.p.) injection [3,4,5].

Saxitoxins—STXs—are a class of alkaloid neurotoxins also known as Paralytic Shellfish Poisons or Toxins (PSP or PST), as they were firstly found in mollusks contaminated with toxic dinoflagellates [6]. There are about 22 variants among the STXs which might have no saxitoxin or neosaxitoxin, only one G-toxin(s) or two C-toxin(s) SO^3−^ group in the molecule [3]. These fast acting toxins have different LD50 (i.p.) for mouse, but the mechanism of action is the same. They block the sodium channel in the neurons, leading to impaired action potential and paralysis of muscles, killing mice in 2–30 min [5,7]. STXs can also cause motor incoordination in fish [8,9] and immobilization in cladocerans [10]. STXs were more studied in marine than in freshwater environment [11,12]. *Cylindrospermopsis* is among the genera that produce STXs in freshwaters, and has been quickly spreading in lakes of North America and Europe in the last 10–15 years [13,14,15,16], as well as in South America [17,18,19,20,21]. However, strains isolated from North America and Europe [22] and from Australia and Thailand [23,24] produce cylindrospermopsin (CYN), while strains isolated in Brazil up to date produce STXs [19,20,25]. Cases of intoxication of humans by STXs have been related to consumption of marine shellfish contaminated with toxic dinoflagellates [11] but no case of human intoxication involving STXs-producer cyanobacteria has been documented to date.

The neurotoxin β-*N*-methylamino-l-alanine (BMAA), a nonprotein amino acid, has recently been found not only in *Nostoc* strains isolated from symbiotic relationships with lichen and host plants of broad taxonomic diversity, but also in all morphological groupings of free-living cyanobacteria from freshwater, brackish and marine environments [26]. This suggests that, given the right conditions, virtually all cyanobacteria may produce the toxin and imposes a serious human health hazard as BMAA could be involved in neurodegenerative diseases such as amyotrophic lateral sclerosis and Alzheimer [27].

The hepatotoxins microcystins (MCs) are the best studied class of cyanotoxins. Although the toxicity of MCs-producer strains was well known since the 1950s [28], the identification and chemical structure were determined only in the 1980s [2]. Nodularins (NOD) and MCs are both hepatotoxic cyclic peptides, with five and seven aminoacids, respectively. There are about 70 types of MCs described up to date, changing basically in the combination of their l-aminoacids [3,29], while there are only seven NOD types so far described [30]. *Microcystis* is the most common bloom-forming cyanobacteria in freshwaters and is involved in most cases of intoxication of wild and domestic animals [31] and human contamination [32,33]. Hepatotoxins kill in 45 min to a few hours (after mice receive an intraperitonial injection), resulting from hemorrhagic shock caused by excess of blood in the liver [5]. They are also protein phosphatases 1 and 2A inhibitors and are considered potent tumor promoters in chronic exposures [34]. 

The cyclic guanidinic alkaloid cylindrospermopsin (CYN) is also classified as a hepatotoxin but has a completely different mechanism of action, being a protein synthesis inhibitor, with a major impact on liver cells, but also in other organs such as kidneys, spleen, intestine, thymus and heart in vertebrates, in agreement with the more general concept of cytotoxicity [4]. Contrary to neurotoxic alkaloids, CYN acts very slowly, taking about 5 to 6 days to kill mice with a LD50 of 200 µg kg^−1^ [3]. It has caused serious health problems in drinking water supplies in Australia [3,4,5]. 

Table 1 shows the main cyanotoxins, the main producing genera, their mechanism of action, LD_50_ (i.p.) in mouse bioassays and main detoxication mechanisms involved in the biotransformation of these compounds.

**Table 1:** Cyanobacterial toxins, the main producer genera, mechanism of action, LD_50_ (i.p.) and detoxication pathways. Modified from Wiegand and Pflugmacher [35].

Toxin	Producer	Mechanism of action	LD_50_(i.p.) µg kg^−1 (a)^	Detoxication
Microcystins (MCs)	*Microcystis*	Inhibition protein phosphatase (PP1 and PP2A)	50→1000 ^(b)^	GST
	*Anabaena*			
	*Plankthotrix*			
Nodularin (NOD)	*Nodularia*	Inhibition protein phosphatase (PP1 and PP2A)	50	GST
Saxitoxins (STXs)	Dinoflagellates:	Binding and blocking the sodium channels in neural cells	8–10	GST
	*Protogonyaulux*			
	*Alexandrium*			
	*Gymnodinium*			
	*Pyrodinium*			
	Cyanobacteria:			
	*Anabaena*			
	*Aphanizomenon*			
	*Cylindrospermopsis*			
	*Lyngbya*			
Anatoxins (ANTX-a)	*Anabaena*	Binding irreversibly to the nicotinic acetylcholine receptors	20–250	Cytochrome P450
	*Aphanizomenon*			GST
	*Cylindrospermopsis*			
	*Plankthotrix*			
	*Oscillatoria*			
	*Microcystis*			
Anatoxin-a(s) (ANTX-a(s))	*Anabaena*	Inhibition of Ach-esterase activity	20	Cytochrome P450
				GST
Cylindrospermopsin (CYN)	*Cylindrospermopsis*	Inhibitor of protein biosynthesis cytogenetic damage on DNA	2100 (24 h)	Cytochrome P450
	*Aphanizomenon*		200 (5–6 days)	
	*Umezakia*			
	*Raphidiopsis*			
	*Anabaena*			
Lipopolysaccarides (LPS)	Cyanobacteria in general	Irritant; causes inflammation in exposed tissues	unknown	Deacylation via lysossomal pathway

^(a)^ Values for mouse bioassays; from Chorus and Bartram [3]; ^(b)^ Values for the different variants of microcystins.

Although a great deal of research has been dedicated to the effects of cyanotoxins to warm-blooded terrestrial animals, the ecological role of these toxins in the aquatic environment remains under debate [35,36]. In fact, the explanation why cyanobacteria have developed the capacity to produce such toxins remains unclear. One of several hypotheses is that they work as an anti-grazing compound, especially against zooplankton [37,38]. However, there is not unequivocal evidence about the chemical defense role of cyanotoxins [39,40,41]. Induction of MCs production by zooplankton provided some evidence on the role of these toxins. Studies carried out by Jang *et al.* [42,43] corroborated the defense hypothesis somewhat, but did not unequivocally separate the factors (*i.e.*, zooplankton media or *Scenedesmus* media) that caused the increase of MCs production. 

Some studies point, instead, to the nutritional deficiency as a more important factor than toxicity in conferring poor growth and survivorship to zooplankton species [44,45,46,47]. Cyanobacteria are generally considered a poor quality food for zooplankton, lacking some essential polyunsaturated fatty acids (PUFAs) and sterols [44,45,47,48,49,50,51]. Some studies, however, have shown that some cyanobacteria are relatively rich in PUFAs such as linoleic and α-linolenic acids [52,53] and that some species of zooplankton exhibit good survival and growth when fed cyanobacteria [54,55,56].

Other factors such as cyanobacteria morphology and predator resistance/tolerance to cyanotoxins can additionally contribute to contrasting effects of cyanobacteria on aquatic organisms. Even if cyanobacteria can be consumed by zooplankton at high rates [57,58], large colonies and filaments may reduce the filtering efficiency of some zooplankton species compromising their energy balance [59,60,61]. Recent studies have shown that not only are some zooplankton species able to co-exist with toxic cyanobacteria and even growth in their presence [61,62], but also that a sensitive species can indeed develop tolerance when pre-exposed to toxic cyanobacteria [63,64,65]. The fact that not all species are affected is, in turn, an argument that weakens the chemical defense hypothesis.

In fact, the separation of all these complicating factors requires a very good expertise and experimental design, which is not an easy task. Few studies have shown good evidence in favor of the chemical defense hypothesis [60,66,67,68,69]. Despite the controversial evidence, toxic cyanobacteria are in fact known to exert several effects on zooplankton as well as on other taxa, as will be described in the following sections.

## 2. Accumulation of Cyanotoxins and Their Effects on Aquatic Invertebrates

Although cyanobacteria and their toxins can exert effects in all taxonomic levels, including bacteria, algae and plants, special attention has been dedicated to the effects on aquatic invertebrates, mainly on zooplankton [70,71,72]. Several studies have shown the accumulation of cyanotoxins in some trophic level, while only a few present evidence of cyanotoxins transferring through the food chain both in the field [73,74,75,76,77,78] and experimentally [79,80,81]. 

Table S1 (Supplementary) presents data of the accumulation of cyanotoxins by several taxa of aquatic invertebrates. Data were obtained from indexed databases (e.g., ISI, Web of Science, ScienceDirect, Medline), as well as from non-indexed bases (e.g., Ph.D. thesis, M.Sc. dissertations and non-indexed articles). Whenever possible, data were expressed in μg g^−1^ DW (many data had to be transformed or calculated using authors’ data) and when not possible they were expressed in the original units. There is a broad variation in toxin concentrations, even if the same taxonomic group or species is considered. This variation results not only from the various methodologies employed in the extraction procedure and in the analyses of toxins, but also from the different exposure modes, source of toxins (*i.e.*, live cells, extracts or purified toxins) and, additionally, from the different ingestion, digestion and detoxification capabilities of the different taxa. To further complicate the comparison, while some studies were carried out with field samples, others were performed in the lab in experimental conditions.

### 2.1. Accumulation and Effects of Hepatotoxins on Invertebrates

**Figure 1:**
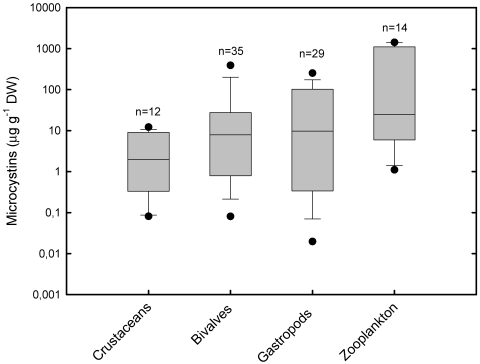
Concentration of hepatotoxins microcystins (MCs) (µg g^−1^ DW) in aquatic invertebrates. Box plots represent median, standard error, 5th and 95th percentile of maximum values found in each study, irrespective of organ/tissue. Black circles represent minimum and maximum values found. Data in crustaceans (Decapoda), bivalves and gastropods are maximum values found in the whole body or organ/tissue (e.g., stomach, liver, muscles, *etc.*), and values in zooplankton are maximum values in the whole body from natural communities or from experimental animals. Field and laboratory samples in the same study were considered as independent samples, regardless of the species. The number of samples (*n*) represents the number of data for each taxa. Data based in a total of 55 studies (not considering the data of Williams *et al.* (ref. 45 in Supplementary Table S1) with Limieux oxidation). Studies were carried out under natural exposure conditions (*i.e.*, blooms or seston samples) or through exposure to cyanobacterial cultures.

As observed for cyanotoxins in general, there is a variation of several orders of magnitude in the concentration of MCs, the most studied toxins, both within and between taxonomic groups of invertebrates (Figure 1 and Supplementary Table S1). In spite of the high variability, zooplankton is clearly the best bioaccumulator of MCs, reaching values over 1000 μg g^−1^ DW and an average value of about 383 μg g^−1^ DW. Gastropods may contain concentrations up to 436 μg g^−1^ DW (*Sinotaia histrica* hepatopancreas [82]), with an average of 58 μg g^−1^ DW. Although bivalves are efficient filter-feeders, maximum concentration was 630 μg g^−1^ DW (*Unio douglasiae* hepatopancreas [83]), and the average was only 57 μg MC g^−1^ DW. Larger crustaceans (*i.e.*, Decapoda) seem to be the less efficient accumulators of MCs, with an average of 4.2 μg g^−1^ DW and a maximum concentration of 12.4 μg g^−1^ DW (*Macrobrachium nipponensis* stomach [84]). 

Even if high concentrations of MCs have been measured in aquatic consumers of different trophic levels, a meta-analysis based on the Biomagnification Factor (BMF) quantitatively confirms biodilution of MCs, not biomagnification [76,85], as the dominant process in aquatic food webs [86].

BMF is herein defined as the ratio between the concentration of MCs in aquatic consumers and the concentration of MCs of their food, both with same unity. Although we will discuss some aspects of BMF in the next sections, here we focused primarily on the potential for accumulation of cyanotoxins and their effects on aquatic invertebrates and vertebrates. For further details on data selection, BMF calculation, analysis and discussion see Kozlowsky-Suzuki *et al.* [86].

Watanabe *et al.* [73] were the first in demonstrating the accumulation of MCs by zooplankton. They found values up to 202% above phytoplankton, but their method of estimation was indirect, using fractionated samples of phytoplankton and zooplankton still contaminated by phytoplankton and calculating the difference in concentration between these samples. The dominant species were the cladocerans *Bosmina falalis* and *Diaphanosoma brachyurum* which have very small body size (~1 μg DW animal^−1^), which makes very difficult to separate from phytoplankton using nets. The amount required for analysis (~10 mg) would require about 10,000 individuals of both species, which would be impossible to separate by trialing animals from the samples. A more powerful detection method, available after their work, the Enzime Linked Immunosorbant Assay (ELISA) allowed the use of very small of biomass (~50–100 µg) [79]. 

Using ELISA, Thorstrup and Christoffersen [79] showed experimentally that *Daphnia magna* was able to accumulate up to 24.5 μg MC g^−1^ DW when submitted to toxic cells of the *Microcystis aeruginosa* strain CYA228/1. These authors also concluded that if the amount of toxins was originated only from the content of the cells in the gut of the animals, calculated by the biovolume as 0.002 ng MC *Daphnia*^−1^, the amount of toxins actually measured (0.19 ng MC *Daphnia*^−1^) was 100 times higher, indicating that this toxins was assimilated and stored in other parts of the animal.

Trophic transfer of MCs in invertebrates has been rarely demonstrated. With a simple food chain model (*M. aeruginosa*→*Daphnia pulex*→*Chaoborus*), Laurén-Määtä *et al.* [80] observed a higher mortality of *Chaoborus* after preying on *Daphnia* fed toxic Microcystis than that fed non-toxic algae, suggesting that *Daphnia* transferred toxins from *Microcystis* to *Chaoborus*. However, as they did not detect MCs in the midge larvae they suggested that most of the toxin was metabolized or excreted in this two-step food chain. 

Ferrão-Filho *et al.* [75] measured up to 16.4 μg MCs g^−1^ DW in zooplankton and estimated BMFs in the order of 0.7 to 43. In comparison, Ibelings *et al.* [76] showed higher accumulation of MCs in zooplankton (up to 1226 μg MCs g^−1^ AFDW), but lower BMF (0.25). In lab experiments, Oberhaus *et al.* [81] showed that *D. pulicaria* was able to accumulate up to 1090 μg MC g^−1^ DW by consuming small filaments of *Planktothrix*. Despite the fact that the above mentioned studies indicated that zooplankton can be an efficient accumulator of MCs and acts as vectors of these toxins to higher levels, few studies found BMF values higher than unity [75,76,77]. 

**Figure 2:**
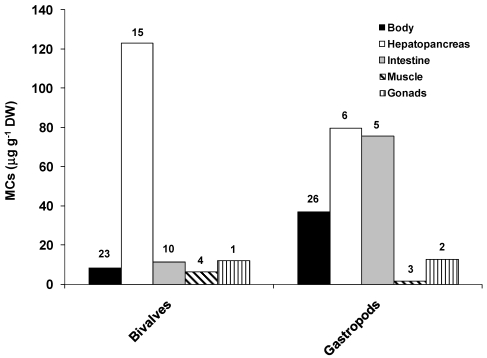
Concentration of MCs (µg g^−1^ DW) in mollusks. Bars represent the mean of maximum values found in each organ/tissue. Numbers on top of bars represent the number of data (n) in each organ/tissue or whole body (Body). Data based in a total of 35 studies (20 for bivalves and 15 for gastropods). Studies were carried out under natural exposure conditions (*i.e.*, blooms or seston samples) or through exposure to cyanobacterial cultures.

Bioaccumulation of MCs in mollusks allows us to study the distribution of toxins in different organs and tissues. Figure 2 shows the mean of maximum concentrations found in organs or tissues of several species of bivalves and gastropods. Hepatopancreas is the organ presenting the highest MCs concentrations, followed by the intestines. Concentrations up to 22.7 μg g^−1^ DW for the gastropod *Sinotaia histrica* [87]. Low concentrations of MCs are, however, found in muscles, and as soon as other organs are removed before food preparation and consumption the risk of contamination is significantly lower. Nevertheless, consumption of mollusks in a regular basis may represent a health threat for humans [88,89,90,91], especially in countries such as China where it is common to consume soup prepared with whole snails [91].

Most studies on NOD accumulation were carried out in the Baltic Sea, mainly in the Gulf of Finland, where *Nodularia spumigena* blooms heavily every year. Both field and laboratory studies estimated the transfer of NOD via zooplankton. Engström-Öst *et al.* [92] verified the bioaccumulation and transfer of NOD in mysidaceans (*Mysia relicta*) and fish (*Gasterosteus aculeatus*) via copepods. Karjalainen *et al.* [93] estimated uptake rates of dissolved NOD of 0.37–1.55 μg gC^−1^ in the calanoid copepods *Acartia tonsa* and *Eurytemora affinis*, and in the ciliate *Strobidium sulcatus*, resulting in bioconcentration factors (BF) of 12, 18 e 22, respectively. Some studies revealed that NOD can be excreted through faecal pellets of copepods [94] and faeces of *Mytilus edulis* [95,96], while others showed that the concentration of NOD in the organisms is positively related with direct consumption of toxic cells [58,96] and transferred to copepods through the microbial food web [97].

Total hepatotoxins including NOD or a NOD-like compound [98] have also been measured in other invertebrates such as the crustaceans *Neomysis integer* [99], *Panaeus monodon* [100], *Gammarus zaddachi* [101] and the bivalve *Macoma balthica* [102]. Although some of these studies indicated trophic transfer of NOD along the food chain, not only low retention of ingested toxins [58] and low toxin concentration of copepod faecal pellets [94] have been observed, but also low bioconcentration of dissolved NOD [93,103] and BMF [87]. In addition, the concentration of NOD in the faecal material of blue mussels can be decreased up to 99% after the process of coprophagy [95] and NOD depuration from grazer tissues takes place rapidly despite incomplete removal [96,104] even after 168 h of cleaning-up period [103].

So far, MCs have been the most extensively studied cyanotoxin regarding its effect on zooplankton [71,105,106,107,108]. MC-producer strains of cyanobacteria may be toxic to a variety of zooplankton species, including rotifers [109,110], cladocerans [71,72], copepods [60,111], and *Artemia salina* [107,112], but the toxicity has hardly been directly related to the production of MCs. Toxicity to zooplankton may, instead, be related to other compounds produced by cyanobacteria. Jungmann and Benndorf [105] isolated a compound from extracts of natural blooms that was more toxic to *Daphnia* than MC-LR and found no correlation between the concentration of MCs from different strains of cyanobacteria and the toxicity to *Daphnia*. Compounds produced by some cyanobacteria that are neither hepatotoxins nor neurotoxins may also cause toxic effects to *Artemia salina* and to the *Aedes aegypti* mosquito larvae [112,113].

Recent studies comparing wild and genetically engineered or spontaneous mutant genotypes of *Microcystis aeruginosa* strains have provided good tests to support the ecological role of MCs [66,67,68,69]. For example, the wild type of the strain PCC7806 is able to produce MCs, while the mutant genotype was engineered to knock out the MC synthetase gene cluster—mcyA-J—and does not produce MCs [66]. Nevertheless, the strain MRC, which has the mcyA-J cluster, is unable to produce MCs [67]. From the same bloom, and sub-cultured from the same original toxic isolate (MR), *M. aeruginosa* strain MRD produces MCs [67]. Thus, the availability of these mutants provides an opportunity to test the hypothesis that the negative effects of *Microcystis* on *Daphnia* growth and reproduction is caused uniquely by the presence of MCs in the cells, because the wild-type and mutant strains only differ in their ability to produce this toxin. Rohrlack *et al.* [66] showed that both ingestion rates and survival were largely inhibited by the wild genotype of the strain PCC7806, while the mutant genotype inhibited ingestion rates but caused no death in *D. galeata*. Kaebernick *et al.* [67] also found higher mortality with the MC-producing mutant (MRD), but ingestion rates were inhibited in a similar manner by both genotypes, showing that MCs were not the factor causing feeding inhibition. These authors found, however, that the wild MC-free genotype (MRC) caused a disruption in the molting process and in the midgut epithelia of two *Daphnia* species. On the other hand, Lürling [68] reported similar growth rates (in body size) for *D. magna* feeding on both genotypes, but a low survivorship with both genotypes, although the wild genotype was a little more toxic. Even though these studies represent good tests of the role of MCs, the incongruence among results suggests that other compounds than MCs may be involved in the toxicity of *M. aeruginosa* to *Daphnia*. For instance, while MCs seem to be the cause of death, the feeding inhibition and disruption in the molting process seem to be caused by a bioactive compound other than MCs.

Rohrlack *et al.* [69] also showed that the wild genotype of the strain PCC7806 caused a reduction in the heartbeats and in the movements of the thoracic limbs, mandibles, and second antennae of *Daphnia galeata* as well as a decrease in the activity of the foregut and stimulation of the midgut muscles. The mutant genotype caused, however, none of these effects. Both genotypes caused, nevertheless, a disruption of the midgut epithelium of *D. galeata*. Hence, all those studies together suggest that MCs can be considered a toxic metabolite to Daphnia, but cannot be considered responsible for all effects observed.

Although the mechanism of action of MCs in invertebrates has been poorly investigated, damage to the cells of the midgut of the *Aedes aegypti* mosquito larvae exposed to MCs [114] and hystopathological alterations in the midgut of *Daphnia magna* exposed to MC-LR [115] and of *D. galeata* exposed to intact cells of toxic *Microcystis* [69] have been demonstrated. DeMott and Dhawale [116] also demonstrated that MC-LR inhibited the activity of protein phosphatases 1 and 2A from bruit extracts of *D. pulex*, *D. pulicaria* and *Diaptomus birgei*, corroborating the results of previous studies in which purified MC-LR and a strain of *Microcystis aeruginosa* (PCC7820) showed acute toxic effects on the same zooplankton species [71]. Biochemical alterations in the activity of other important enzymes have been also demonstrated, such as the inhibition of glutathione (GSH) and glutathione-*S*-transferases (GST) in *D. magna* [115], tripsyns and chymotrypsins in *Moina macrocopa* and *D. magna* [117,118], soluble GST in *Artemia salina* [107], microsomal GST in *D. magna* [119], acetylcholinesterase (AChE) in *D. pulicaria* [120] and a stimulation of the enzyme lactate-dehydrogenase in *Daphnia magna* [115].

MCs and toxic cyanobacteria can also exert effects on other aquatic invertebrates such as crustaceans, gastropodes and insect larvae. The standard test with the anostracoid crustacean *Tamnocephalus platyurus* has been applied in some studies in a tentative to verify the toxicity of cyanobacterial blooms, providing results comparable to mouse bioassays [121,122,123,124,125]. Vasconcelos *et al.* [126] tested the effects of living cells of toxic and non-toxic strains of *M. aeruginosa* on *Procambarus clarkii* (Crustacea, Decapoda) and showed that juveniles were more susceptible to non-toxic strains, suggesting that other toxins or compounds, more potent than MCs, may have being produced. Montagnolli *et al.* [127] tested the toxicity of extracts of a MC-producer strain of *M. aeruginosa* on *Kalliapseudes schubartii* (Crustacea, Tanaidacea) and estimated a LC_50_ (96 h) of 1440 mg L^−1^ (equivalent to a 1.58 mg MC L^−1^), and an increase in the oxygen consumption. Pinho *et al.* [128,129] studied the response of hepatopancreas and gills of the crab *Chasmagnathus granulatus* (Decapoda, Brachyura) to the oxidative stress after exposure to MC-aqueous extracts, through the activity of catalase (CAT), superoxidase dismutase (SOD) and glutatione-*S*-transferase (GST), as well as with measures of oxygen (O_2_) consumption, formation of sulfidril groups (NP-SH) and lipid peroxides (LPO). They observed increase in the consumption of O_2_, and in the activity of CAT and GST, as well as oxidative damage by the increase in LPO. In addition, extracts of *M. aeruginosa* administered orally on the same crab caused a decrease of the content of glycogen, which indicates that the increase in O_2_ consumption may be associated to the glycolitic pathway [130].

Maršálek and Bláha [124] tested the toxicity of crude extracts and fractions of purified toxins from natural assemblages of *M. aeruginosa* with a series of aquatic invertebrates such as the protozoans *Tetrahymena pyriformis*, *T. termophyla* and *Spirostomum ambiguum*, the shrimp *Artemia salina*, the cladocerans *D. magna*, *D. pulex* and *Ceriodaphnia dubia*, the anostracoid crustacean *T. platyurus*, the rotifer *Brachionus calyciflorus*, the nematode *Panagrellus redivivus*, the *Culex pipiens* mosquito larvae, the cnidarian *Hydra tenuata* and the oligochaeta *Tubifex tubifex*. However, there was a large variability in the sensitivity of these organisms and results were not always correlated with MC concentration. For some invertebrates, such as *A. salina*, *P. redivivus*, *T. pyriformis* and *T. platyurus* there was a significant difference in the toxicity of crude extracts and corresponding toxin fractions (*i.e.*, crude > toxin) while for all others no significant differences in the toxicities of both extract preparations (crude *vs.* toxin fraction) were found.

Mosquito larvae, such as that of *Aedes aegypti* also show sensitivity to cyanobacteria [113,114,131]. For example, a MC-producing strain of *M. aeruginosa* led to significantly longer development times than the controls or those grown with the non MC-producing strain [131].

Despite their high resistance to cyanotoxins, mollusks may also be affected by these compounds. The pulmonate gastropod *Lymnaea stagnalis*, besides accumulating MC-LR, presented a reduction in the egg production and in locomotion when exposed to purified toxin [132]. The zebra mussel, *Dreissenia polymorpha*, may show a large reduction in the food ingestion and absorption rate, leading to a reduction in the net energy balance, especially when exposed to single diets or mixtures of a MC-LF-producing *M. aeruginosa* and the diatom *Asterionela formosa* [133]. Toxic cells may also cause an acute irritant response manifested by the production of an unusually fluid pseudofaeces (“pseudodiarrhoea”) rich in mucus and *Microcystis* cells, ejected through the pedal gape of the mussels [134]. This selective rejection of MC-producing cyanobacteria by zebra mussels may enhance the presence of toxic *M. aeruginosa* in mixed cyanobacterial blooms and in the benthos [133].

The effects of NOD and/or toxic *N. spumigena* have been studied on different invertebrates. Koski *et al.* [57] studied the feeding and egg production of calanoid copepods (*Acartia bifilosa* and *Eurytemora affinis*) treated with water from a mesocosm experiment containing *N. spumigena*. Although copepods preferred ciliates, significant amounts of cyanobacteria were consumed with no adverse effect in the survivorship and egg production. Despite high ingestion rates by *A. bifilosa* and *E. affinis* on toxic *N. spumigena* offered alone or in mixtures with a green flagellated alga (*Brachimonas submarina*) or natural community, gross growth efficiency was low suggesting a high metabolic cost of coping with toxins [58].

Karjalainen *et al.* [135] reviewed the effects of Baltic Sea cyanobacteria including hepatotoxic *N. spumigena* on aquatic organisms including invertebrates. More recent studies have observed no apparent negative effect of *N. spumigena* on the survival of Baltic Sea meiofaunal assemblages under experimental conditions [136]. In fact, cyanobacterial labelled carbon was taken up by major meiofauna taxa living in the first centimeter layer indicating that cyanobacteria may be an important food source for the Baltic Sea meiobenthos. Nevertheless, even if cyanobacterial carbon can for instance be assimilated by the amphipod *Monoporeia affinis*, this does not translate into significant growth [137]. These authors also observed that less cyanobacterial carbon from toxic *N. spumigena* was mixed down in the sediment indicating lower bioturbation activity. 

### 2.2. Accumulation and Effects of Cylindrospermopsin on Invertebrates

Evidence on CYN accumulation in aquatic invertebrates is scarce and a broader review of the bioaccumulation and effects of this toxin can be found in Kinnear [138] (this volume). *Cherax quadricarinatus* (Decapoda) from aquaculture ponds and in laboratory conditions may accumulate CYN in higher concentrations in the hepatopancreas [139] (Table S1). Saker *et al.* [140] found CYN concentrations of 2.9 and 5.9 μg g^−1^ in whole body and viscera, respectively, of the bivalve *Anodonta cygnea*, but higher values (61.5 μg g^−1^) in the haemolymph, corresponding to 408 μg CYN L^−1^, when the concentration in the medium was 34 μg CYN L^−1^, which gives a bioaccumulation factor (BF) of 12. In both studies, no adverse effects were observed. Despite toxin transfer, low BF (0.41–0.76) of CYN in relation to the surrounding medium was estimated for *D. magna* suggesting no evidence biomagnnification [141]. In the same study, *D. magna* was exposed to a CYN-producer strain (Cylin-A) and to a non CYN-producer strain (Cylin-P) of *Cylindrospermopsis racibosrkii* and both strains reduced the survivorship and growth, with the Cylin-A being more effective, suggesting that CYN was the toxic agent. In another study, however, two CYN-producer strains (*C. raciborskii* and *Aphanizomenon ovalisporum*) caused a reduction in the survivorship and growth of *D. magna*, but only the *C. racibosrskii* strain caused damage in the midgut and diverticula epithelium [142]. This result suggests that not all adverse effects on *Daphnia* can be attributable to CYN.

White *et al.* [143] detected CYN bioconcentration and bioaccumulation in *Melanoides tuberculata* (Gastropoda) after exposure to freeze-thawed cell extracts and live cells of toxic *C. racibosrskii*, with average BF and bioaccumulation (BAF) factors up to 1.5 and 124.4, respectively. Exposure to live cells resulted in higher toxin burden (up to 250 µg g^−1^ DW) than exposure to freeze-thawed solutions indicating that despite uptake from both extracellular and intracellular pathways, the availability of intracellular toxin is critical. Berry and Lind [144] presented evidence for CYN bioaccumulation in a freshwater lake in Mexico. They measured appreciable concentrations of both CYN and STXs in “tegogolo” snails (*Pomacea patula catemacensis*), despite low concentrations in the seston, and calculated BAFs of 157 and 196, respectively. These studies suggest that CYN and STX may have a higher (or at least equal) potential for biomagnification than MCs.

### 2.3. Accumulation and Effects of Neurotoxins on Invertebrates

Contrary to the vast literature from marine studies [11], there is very little evidence of bioaccumulation and effects of STXs produced by cyanobacteria in freshwaters. Sasner *et al.* [145] was one of the first studies showing the accumulation of STXs (PST) produced by *Aphanizomenon flos-aquae* in the bivalves *Ellipio campanatus* and *Corbicula fluminea*. Negri and Jones [146] showed the accumulation of STXs produced by the cyanobacterium *Anabaena circinalis* in the mollusk *Alathyria condola*. They reported an “adverse effect on feeding behavior” of the mollusks but did not quantify any physiological parameter. Nogueira *et al.* [147] showed not only that *D. magna* accumulated STXs from *Aphanizomenon issatschenkoi*, but also that both fitness and somatic growth of this daphniid were negatively affected, and that the activity of the cytosolic glutathione-S-transferases (cGSTs) decreased. Although STXs detoxification via GST activity is possible [35], there is no available data about the conjugation of SXTs and GSH. 

Pereira *et al.* [148] estimated the accumulation of PST from *A. issatschenkoi* in the bivalve *Anodonta cygnea* and their BMF estimates varied from 0.035 to 2.2, thus in the range observed for MCs [86]. PST also affected the filtering behavior of mussels, though low mortality rates were observed.

Few studies have demonstrated the effects of STXs in freshwater zooplankton species. Reduction in the thoracic appendages beating rate and an increase in the rejection rate of particles by the post-abdomen of *Daphnia carinata* was observed after exposure to a filtrate of *Aphanizomenon flos-aquae* and purified STX [149]. Also, the intrinsic rate of population increase (*r*) of *D. pulex* and *Moina micrura* was negatively affected by a STXs-producer strain of *C. raciborskii* (T3), but not by a non STXs-producer strain (NPLP-1) of the same species [150]. In contrast, *D. gessneri* was stimulated by strain T3 and depressed by the strain NPLP-1, suggesting resistance to STXs produced by the strain T3 and sensitivity to some bioactive compound(s) produced by the strain NPLP-1. *D. magna* had slightly longer survivorship in suspensions with another saxitoxin-producer strain of *C. raciborskii* (CYRF-01) as the sole feed than in medium without food, showing that starvation was not the cause of death [151]. However, these authors showed that the fitness (*i.e.*, body growth and population growth rates) and clearance rates were depressed only in high proportions (75–100%) of this cyanobacterium in the diet, and concluded that energy limitation, not toxicity, might be the dominant factor affecting growth of large-bodied cladocerans. These contradictory results exemplify how generalizations on cyanobacteria-zooplankton interactions are prone to failure and that species-especific interactions may occur.

STXs can also affect the swimming behavior of zooplankton. Using an image analysis system to track the swimming activity of *Daphnia* exposed to a STXs-producer *C. raciborskii* strain (CYRF-01) and to raw water samples from a eutrophic reservoir, Ferrão-Filho *et al.* [152] showed a decrease in the mean time spent in swimming and in the mean velocity of *D. pulex*, which was attributed to the presence of STXs in field samples and in the strain CYRF-01. Swimming movements of *D. pulex* and *M. micrura* were also inhibited by the strain T3 in 24 h exposures, but recovered within 48 h after the animals were transferred to a medium with only highly nutritious food [10]. A recent study [153] has, however, shown that both paralysis and recovery can occur even faster (paralysis in less than 1 h and recovery in less than 24 h). Cladocerans basically swim by the movements of the second antennae, thus it is likely that STXs are acting by the classic mechanism of action (*i.e.*, blockage of sodium channels in the neuro-muscular junction), preventing the propagation of the nerve impulse in this appendage. Apparently, the filtration of food particles was not altered allowing the animals to survive for long periods [10]. This curious phenomenon was also observed by Costa [150] with the T3 strain during chronic exposures. Animals could even grow and reproduce, though in a lower rate, while paralyzed on the bottom of the test tube. It is unclear how those animals cope with paralysis of the second antennae while keeping the movement of the thoracic appendages.

Fewer studies were performed with other neurotoxin-producer cyanobacteria. Living cells of an ANTX-a(s)-producer strain of *Anabaena flos-aquae* (NRC525-17) had acute toxic effect on the copepod *Diaptomus birgei* and on the cladoceran *D. pulicaria* [71]. Another ANTX-a(s)-producer strain of *A. flos-aquae* (IC-1) was tested on rotifer species, being *Brachionus calyciflorus* the most susceptible with suppressed fecundity at concentrations of 0.5 mg DW L^−1^ of living cells [154]. Acute toxicity tests with living cells and extracts of lyophilized cells of an ANTX-a(s)-producer strain of *A. spiroides* (ITEP-024) were carried out with *D. gessneri*, *D. pulex* and *Moina micrura*, but little effect was observed [150].

High concentrations of BMAA in aquatic food webs from South Florida in water-bodies where cyanobacteria bloom have recently been found [155]. However, unlike the biomagnification pattern observed for a terrestrial food web [27], no tendency for biomagnification was detected. They observed, instead, higher levels of BMAA in invertebrates such as pink shrimp (*Panaeus duorarum*; ~3000 µg g^−1^ WW) and blue crabs (*Callinectes sapidus*; ~7000 µg g^−1^ WW), and fish species feeding on benthos than those with planktonic feeding habit. Nevertheless, the still scarce knowledge about BMAA indicates the potential for biomagnification with serious implications for human contamination and association with neurodegenerative diseases.

## 3. Accumulation of Cyanotoxins and Their Effects on Aquatic Vertebrates

The accumulation of cyanotoxins and effects of cyanobacteria on vertebrates have focused mostly on fish, the main secondary and tertiary consumers in aquatic food webs (Supplementary Table S2). Other studies have reported effects on amphibians [156,157,158], on turtles [159,160] and birds [160,161,162,163,164,165,166]. 

### 3.1. Accumulation and Effects of Hepatotoxins on Fish

**Figure 3:**
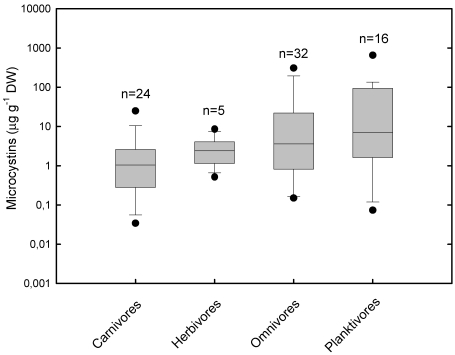
Concentration of MCs in fish by trophic guild. Box plots represent median, standard error, 5th and 95th percentile of maximum values found in each study, irrespective of organ/tissue. Black circles represent minimum and maximum values found. Field and laboratory samples in the same study were considered as independent samples, regardless of the species. The number of samples (*n*) represents the number of data in each guild. Data is based on a total of 28 studies. Studies were carried out in one of following conditions: natural conditions (blooms or seston samples), exposure to dissolved toxins or to experimental pellets containing toxic food or to cyanobacterial cultures. Unnatural exposure routes (e.g., force-feeding, i.p. injections) were not included.

Despite large data variability (Figure 3), the potential for accumulation of MCs in fish seems to be lower than for invertebrates, being on average 3.5 times lower in planktivores than in zooplankton (Figure 2 and Figure 3). The highest concentrations were found in planktivorous fish reaching up to 874 µg MC s g^−1^DW (liver of smelt *Osmerus eperlanus* [76]). Other planktivorous fish, especially carp that feeds on phytoplankton cells, have also a great potential for MCs accumulation. Xie *et al.* [167] found up to 137 µg MC g^−1^ DW in the intestines of silver carp (*Hypophthalmichthys molitrix*) from Lake Chaohu, in China. The maximum concentration of a carnivorous fish was 51 µg MC g^−1^ DW in the liver of perch (*Perca fluviatilis*) of Lake IJsselmeer in the Netherlands [76].

Overall carnivorous fish, as top predators, had lower average MC contents than other trophic guilds (Figure 3), which suggests no tendency of biomagnification along the food chain. In fact, Ibelings *et al.* [76] showed generally low BMFs for a real food chain in Lake IJsselmeer, in the Netherlands. The BMF for the planktivorous smelt reached, however, 286% of its diet (*i.e.*, MCs content of planktivores were ~2.9 times higher than that of zooplankton), while the BMF for the following trophic level (planktivores→carnivores) was only 11%. Surprisingly, the benthivorous ruffe (*Gymnocephalus cernua*), which feeds on pseudofaeces of the zebra mussel (*Dreissena polimorpha*), accumulated high amounts of MCs (up to 194 µg g^−1^ DW), reaching a BMF of 1.2. Furthermore, both a qualitative assessment [85] and meta-analyses of a large dataset on aquatic consumers of different trophic levels and guilds [86] pointed out to biodilution, not biomagnification, of MCs as the predominant process in aquatic food webs. However, zooplanktivorous fish was an exception to this pattern having the highest BMF mean (1.8) among fish guilds, suggesting possible biomagnification of MCs [86]. 

**Figure 4:**
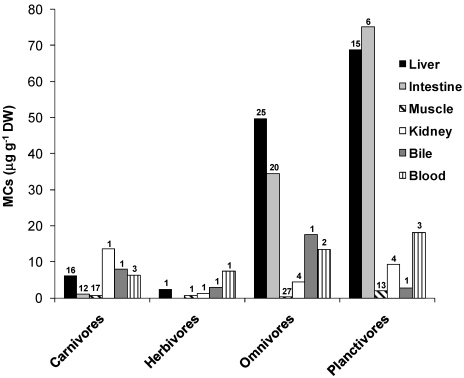
Concentration of MCs (µg g-1DW) in fish organs/tissues by trophic guild. Bars represent the mean of maximum values found in each organ/tissue. Numbers on top of bars represent the number of data (*n*) in each organ/tissue. Data based in a total of 28 studies (12 for carnivores, 1 for herbivores, 19 for omnivores and 13 for planktivores). Studies were carried out in one of following conditions: natural conditions (blooms or seston samples), exposure to dissolved toxins or to experimental pellets containing toxic food or to cyanobacterial cultures. Unnatural exposure routes (e.g., force-feeding, i.p. injections) were not included.

The concentrations of MCs in the different organs/tissues by fish guild (Figure 4) reveal that, as predicted for hepatotoxins, the liver of planktivores (0.06–874 µg g^−1^ DW); and omnivores (0.003–321 µg g^−1^ DW) accumulates most of these toxins, followed by intestines (Planktivores: 5.4–137 µg g^−1^ DW; Omnivores: 0.03–678 µg g^−1^ DW). Other tissues such as kidney, bile and blood can also accumulate relatively high concentrations of MCs [168]. Low concentrations of MCs can be found in the gills of carnivores (up to 0.1 µg g^−1^ WW), omnivores (up to 1.4 µg g^−1^ WW) and planktivores (0.56 µg g^−1^WW) [170]. Papadimitriou *et al.* [169] found low concentrations of MCs in the brain (0.044 µg g^−1^ WW) and ovaries (0.009 µg g^−1^ WW) of the omnivorous fish *Carassius gibelio* in Greek lakes. Despite low concentrations of MCs in fish muscles (up to 14.3 µg g^−1^ DW), great concern is given to this tissue as it is part of human diet in many countries. The World Health Organization (WHO) has established guidelines for the consumption of fish containing MCs. A Tolerable Daily Intake (TDI) value of 0.04 µg MC kg^−1^ of body weight day^−1^ was adopted as a provisional guideline value [3]. Magalhães *et al.* [170] found a maximum daily intake of 1.68 µg MC kg^−1^ in fish from Jacarepaguá Lagoon, which is 42 times above the proposed TDI. The authors pointed out that this form of human exposure must be monitored, and that the quality control of fish from water bodies presenting cyanobacterial blooms should be carried out.

Few experimental studies showed that primary consumers may act as an efficient vector of toxins to upper trophic levels such as fish. Pumpkinseed sunfish (*Lepomis gibosus*) accumulated MCs (up to 11.2 ng MC g^−1^ WW and only 250 pg MC g^−1^ WW in liver and muscles, respectively) from lyophilized zooplankton from the eutrophic Barbaboes Pond (New Hampshire, USA) [171]. Transfer of free and bound MCs from intoxicated snail *Lymnaea stagnalis* to stickleback *Gasterosteus aculeatus* has been shown, with MCs accumulating in fish liver, kidneys, gills and muscle [172]. Induction of oxidative stress, liver histopathology and behavioral disturbances were also observed [172]. 

NOD or a NOD-like compound has been measured in different fish species of the Baltic Sea, such as flounder and cod [173], sea trout [174], pike larvae [99], roach [165], three-spined stickleback, herring and sprat [175] from field or experiment samples. Persson *et al.* [176] provided the first data on NOD accumulation in flounder liver tissue from the Öresund (Swedish west coast) despite non-contaminated primary food source (blue mussels).

Although mass mortality of fish may be related to harmful algal blooms [177,178,179,180] factors such as high pH due to photosynthetic activity and low O_2_ concentrations associated to the decay of algal biomass, may as well be related to these events [35]. Toxic cyanobacteria or their toxins may, nevertheless, exert adverse effects on fish, including damage to several organs such as liver, heart, gills and kidneys, besides ionic equilibrium disturbance, behavioral changes, growth inhibition and mortality [181]. However, as observed for other aquatic organisms results are generally non-conclusive and contradictory. For instance, Beveridge *et al.* [182] showed an increase in the opercular beating rate of the Nile tilapia (*Oreochromis niloticus*) and an increase in the ingestion rate of cyanobacteria with a non-toxic strain of *M. aeruginosa* but no effect with a toxic strain (MC-LR-producer), whereas Keshavanath *et al.* [183] observed an inverse response for the same fish species. The European roach *Rutilus rutilus* fed with *Aphanizomenon* or *Microcystis* cells collected from eutrophic lakes presented a low growth rate [184]. Nevertheless, growth rate with *Aphanizomenon* was significantly higher than without any food and low growth with *Microcystis* could not be attributed to the presence of MCs, but to the low digestibility of this cyanobacterium and low assimilation efficiency by the roach [184].

Best *et al.* [185] found that the rainbow trout (*Oncorhynchus mykiss*) exposed to intact or broken cell extracts of MCs-producer *Microcystis* (PCC 7813) had an increase in liver mass (hepatosomatic index, HSI) and in the water content in the gut, but only when administered together with heterotrophic bacterial lipopolysaccarides (LPS). They concluded that exposure of fish to toxic *Microcystis* promoted osmoregulatory imbalance resulting from stimulation of the drinking response, increased volume of fluid in the gut and inability to remove water excess. Bacterial LPS is known to affect numerous systems, including the liver detoxication system [186,187] and it is likely that cyanobacterial LPS exhibits similar effects to their bacterial counterparts [188], including the inhibition of glutathione *S*-transferases [189].

Ibelings *et al*. [76] reported histological abnormalities such as inflammation, degeneration and necrosis, potentially caused by MCs, in 50% of the smelt livers in Lake IJsselmeer. For the other species, especially ruffe, this percentage was lower. However, none of the livers were enlarged or darker as was observed in gavage experiments with perch exposed to MCs. 

Histopathological alterations in liver, gastrointestinal tract and kidney, and also immunopositivity for MCs, heve been observed in the European whitefish *Coregonus lavaretus* when administered an oral dose (*i.e.*, gavage) of a MC-producer strain culture of *Planktothrix rubecens* [190], suggesting a relationship between tissue damage and the presence of MCs. In addition, exposed fish showed behavioral alterations, such as increased opercular beat rates and elevated plasma glucose levels, possibly representing a physiological stress response. In another study, the same histopatological changes in the liver, kidney, and in the gastrointestinal tract of *C. lavaretus* sub-chronically exposed to ambient water containing low, medium and high *P. rubescens* densities were found [191]. However, gill pathology appeared to result primarily from mechanical abrasion and irritation due to ectoparasitic infestation. These studies confirmed the hypothesis that sub-chronic and chronic exposure to low cyanobacterial cell densities and hence MCs can exacerbate physiological stress and sustained pathological alterations in exposed coregonids. They also corroborated the evidence that *P. rubescens* blooms may be the cause of the observed weight reduction (*i.e.*, fitness) of this fish in European lakes.

Liver histopathology has been examined in sea trout [174] and flounder [192,193] either from Baltic Sea samples or experimentally after oral administration of toxic *N. spumigena* containing slurry. Flounder liver histopathology from field specimens had no connection with toxin concentrations and many changes and lesions were likely related to the presence of parasites or caused by combined factors such as various anthropogenic pollutants [100,192]. Both flounder [193] and sea trout [174] liver were affected after oral administration, but the effect on sea trout liver was more severe and liver architecture was completely lost after 1–2 days after a single dose despite low ELISA signal. Sea trout liver partially recovered after 4–8 days, indicating that in spite of the apparent high NOD toxicity, the effect was severe but reversible.

Effects of hepatotoxins or toxic cyanobacteria at the biochemical level were also observed in fish. Pflugmacher *et al.* [194] found a reduction in GST activity with MC-LR in several organisms, including fish. The uptake of MC-LR in larval stages of *Danio rerio* increased the activity of two detoxification enzymes (GST e glutatione-peroxidase, GPx), probably indicating an ability of this organism in metabolizing MC-LR to a less toxic compound [195]. Therefore, chronic effects such as growth inhibition in embryonary stages, when organogenesis is still not completed could be caused by energetic demands of the detoxification process [195]. Bury *et al.* [196,197] observed, however, that an extract of *Microcystis* inhibited the ionic pump of the gills of tilapia (*Oreochromis mossambicus*) more effectively than purified MC-LR, showing that this effect was due to fatty acids present in cells of *Microcystis*, which interact in the membranes of the gill epithelium. 

Using intraperitoneal injections of MC-LR and MC-RR, Prieto *et al.* [198] studied the response of the antioxidant enzymes superoxide dismutase (SOD), catalase (CAT), GPx and glutathione reductase (GR) as well as lipid peroxidation (LPO) as a biomarker of oxygen-mediated toxicity in liver, kidney and gill of tilapia (*Oreochromis* sp.). Their results showed that MCs induce adaptive responses such as increased activity of the antioxidant enzymes, mainly SOD and CAT, as well as of LPO. Regarding LPO, the liver was the most affected organ by MC-LR, but was not affected by MC-RR.

Cazenave *et al.* [199] assessed the oxidative stress in embryos of zebra fish (*D. rerio*) to the exposure to purified MC-LF and MC-RR by determining both the production of hydrogen peroxide and the activity of the antioxidative enzymes guajacol peroxidase (POD), CAT, GPx, and GR. Energetic costs were evaluated by determining the content of fat, carbohydrates, and proteins in exposed and control embryos. They evaluated also the attenuating effect of exposures in combination with natural organic matter (NOM). They verified less pronounced teratological effects within 24 h as well as non-significant increase in the activity of sGST in embryos exposed to either MC-LF or MC-RR in combination with organic matter compared to embryos exposed to pure toxins. NOM also diminished oxidative effects caused by MC-LF, while it failed to attenuate oxidative stress caused by MC-RR. Energetic costs of detoxification were detected by a significant decrease in lipid content, especially in embryos exposed to pure toxins, following a similar trend to that obtained with teratological and enzymatic assays, confirming the attenuating effect of NOM. They concluded that physiological responses to MCs and NOM required energetic costs, which were compensated to the expense of the energy resources of the yolk, which in turn might affect the normal development of embryos.

Responses of the antioxidant enzymes GST, CAT and the formation of malondialdehyde (MDA) in liver tissue of flounder (*Platichthys flesus*) were monitored during 14 days after two doses of intraperitoneal injection of pure NOD [176]. GST and CAT activity decreased significantly in the highest concentration (50 µg NOD kg^−1^) after 7 days, restoring to control levels after 10 days of recovery. No significant changes were, however, seen in MDA levels. Flounders have also been orally exposed to toxic *Nodularia spumigena*-containing slurry either as a single high dose or as three repeated lower doses 3 days apart [193]. Liver GST activity of flounders that received repeated doses was significantly higher than with single dose or control. This result together with a lower NOD concentration in the liver of the repeatedly dosed individuals suggest that repeated doses may increase detoxification efficiency, thus NOD is rapidly detoxificated.

Behavioral changes were also reported in fish exposed to hepatotoxins. Baganz *et al.* [200] showed that the swimming activity (*i.e.*, the average swimming velocity and the average number of turns) of *D. rerio* and *Leucaspius delineatus* exposed to MC–LR presented a dose-dependent response, but that it was dependent on the time of the day (daytime or night-time). During the day, the activity of both species increased in the lower concentrations, while the higher concentrations led to a significant reduction in activity. These results suggest both fish have comparable susceptibility to MC-LR and may indicate some adverse consequences for fish populations.

### 3.2. Accumulation and Effects of Cylindrospermopsin on Fish

Saker and Eaglesham [139] were the first to show CYN accumulation in fish (viscera of the Rainbow fish; *Melanotaenia eachamensis*) from an aquaculture pond in Australia (ref. 22 in Supplementary Table S2). Since then, few studies about CYN accumulation in aquatic vertebrates have been published (see Kinnear [138] in this volume for a complete review on CYN accumulation).

Berry *et al.* [201] tested the effects of purified CYN and extracts of CYN-producer *C. raciborskii* and *A. ovalispurum* on zebrafish. They found that purified CYN was toxic only when injected directly into embryos, but not by direct immersion at doses up to 50 mg L^−1^. Despite the dose dependency of toxicity after injection of CYN, no consistent patterns of developmental alterations were observed, suggesting that toxic effects of CYN may not target specific developmental pathways. In contrast, direct immersion of embryos in all of the extracts resulted in both increased mortality and developmental dysfunctions, such as undeveloped eyes, truncated body axis, severe oedema and curved spine. Interestingly, there was no correlation of developmental toxicity observed for these extracts with the presence of CYN or with previously reported toxicity for these strains. These results suggest that CYN is lethal to zebrafish embryos, but apparently inhibits no specific developmental pathways, whereas other unknown metabolites from *C. raciborskii* and *A. ovalisporum* may be involved in developmental dysfunctions in zebrafish. 

### 3.3. Accumulation and Effects of Neurotoxins on Fish

Evidence of the accumulation and effects of cyanobacterial neurotoxins on fish is still scarce. Osswald *et al.* [202] was the only study that showed the accumulation of ANTX-a in juveniles of the common carp (*Cyprinus carpio*) exposed to freeze-dried cells of *Anabaena* sp. (ANA 37), calculating a maximal theoretical bioaccumulation factor (BAF) of 2.65. They also observed a high lethality in concentrations of 10^7^ cells mL^−1^, with 100% mortality after 26–29 h exposure. In another study, Osswald *et al.* [203] exposed embryos of the same fish to extracts of the same *Anabaena* strain and pure ANTX-a and verified the effects on several parameters of the developmental stages such as time to mortality, mortality rate, time to hatching, hatching rate, skeletal malformations rate, and larval standard length. At all tested concentrations of ANTX-a, the pure toxin was almost harmless to carp early stages of development, contrarily to cell extracts that were highly toxic. Only an adverse effect on the larval length was found at the highest concentration of pure toxin, while increasing concentrations of cell extracts caused increasing adverse effects in all parameters. Authors argued that other bioactive substances could be responsible for the extracts toxicity such as the lipopolysaccharides (LPS), mueggelone and lupenyl acetate, all found in strain ANA 37. 

Oberemm *et al.* [204] reported that pure STX delayed hatching of zebrafish embryos and led to malformations (*i.e.*, lateral and ventral body curvature and edema) and mortalities, while ANTX-a altered the heart rate in zebrafish, but no chronic effects were observed. Compared to results with pure toxins, much more pronounced effects were observed inembryos when exposed to crude aqueous extracts of cyanobacteria from field samples and batch cultures, but based on the toxin analysis they concluded that these effects cannot be explained by known toxins alone. Therefore, these studies emphasize that, even when using ecologically relevant toxin concentrations, the effects of pure toxins do not mimic the effects in the natural environment. The synergistic actions of different toxins and of toxins in complex matrix samples may increase toxicity considerably.

Clemente *et al.* [205] found GTX variants in fish muscle (0.012–0.02 µg STX eq. g^−1^ WW) of *Geophagus brasiliensis* in Alagados Reservoir (Southern Brazil), along with histopathological and several biomarkers alterations such as LPO, genetic damage (comet assay), GST, AchE and EROD activities. In experiments with the carnivorous fish *Hoplias malabaricus*, Silva *et al.* [206] offered contamined prey (*Astyanax* sp.) that were intraperitonealy inoculated with a lysate of *C. raciborskii* culture containing PSP constituted by 97% STX, 3% neosaxitoxin (NeoSTX) plus gonyautoxin (GTX2). Although they did not find any PST analogue in fish muscle, the activity of the enzyme SOD increased, suggesting the generation of free radicals, whereas the activities of CAT, GST and GPx decreased, suggesting an inhibition of the antioxidant system in the brain. In addition, lipoperoxidation (LPO) and protein carbonylation (PCO) increased along with damages in DNA (comet assay), suggesting the occurrence of oxidative stress, genotoxic damage and apoptotic processes in fish exposed to STX.

Along with the biochemical and physiological effects, neurotoxins may cause behavioral changes in fish. Lefebvre *et al.* [9] showed that STXs can alter the sensorimotor function of herring (*Clupea harengus pallasi*), decreasing its response to spontaneous and touch-activated swimming behavior. However, the normal motor function recovered after 4–24 h of continuous exposure. In contrast, Ferrão-Filho *et al.* [10], showed that *D. rerio* exposed to a STXs-producer strain of *C. raciborskii* (CYRF-01) and to water samples from a eutrophic reservoir containing this and other cyanobacteria, had a significant increase in swimming activity (*i.e.*, mean distance performed and mean velocity). The result obtained by Lefebvre *et al.* [9] is more compatible with the mechanism of action of STXs (*i.e.*, paralysis), suggesting that other toxins or compounds, which may have irritant properties (e.g., LPS), probably in contact with the gills, might be involved in the stress response of *D. rerio* [10]. Osswald *et al.* [202] showed that intact cells of an ANTX-a producer strain of *Anabaena* caused an increase in the opercular movement and abnormal swimming of juveniles of the common carp (*Cyprinus carpio*). Despite contradictory results, all these effects on fish behaviour by neurotoxic strains may have negative consequences on the fitness of fish populations by changes in reproductive aspects and on predator—prey interactions.

### 3.4. Effects of Cyanotoxins on Other Aquatic Vertebrates

Cyanobacteria may also cause adverse effects to other aquatic vertebrates. Oberemm *et al.* [204] tested the effects of purified MCs, STXs and crude extracts of cyanobacterial strains on the caudate amphibians axolotl (*Ambystoma mexicana*) and smooth newt (*Triturus vulgaris*), and on marsh frog (*Rana ridibunda*). Although no effects of MCs were observed during short-term embryonic development test for all amphibian species, prolonged exposure (35 days) of axolotl to the highest concentration of MC-LR resulted in less developed forelimbs of larvae. STX caused no acute effects on axolotl, but hatching started slightly earlier at lower STX concentrations (10 µg L^−1^), whereas higher concentrations (50–500 µg L^−1^) led to a minor delay in hatching. Crude extracts, however, lead to much more pronounced effects. Extracts of *Anabaena floes-aquae* (ANTX-a producer), for example, caused a dorsal curvature of the axolotl’s tail, along with cranio-facial malformations and gill hyperplasia. 

Dvořáková *et al.* [156] studied the effects of purified MC-LR and samples of natural water blooms of *Microcystis* that contained or not MCs on the African clawed frog (*Xenopus laevis*) embryos in a 96 h teratogenesis assay (FETAX). They showed that pure MC-LR caused weak lethality at the highest concentration tested (LC_25_ = 380 µg L^−1^) and that cyanobacterial biomass caused significant lethality to frog embryos, but biomass containing MC-LR had a higher lethality (LC_25_ = 125 mg DW L^−1^ or 103 µg MC-LR L^−1^) compared to biomass without MC-LR (LC_25_ = 232 mg DW L^−1^). Purified MC-LR as well as both samples of biomass (with and without MC-LR) induced frequent developmental abnormalities in the spinal area (backbone, tail) and malformations of other organs, such as the cranio-facial segment (head, brain, and eyes). Purified MC-LR, however, induced more frequent improper gut coiling and malformations in the abdomen and occurred even at very low concentrations (10–25 µg L^−1^). The teratogenic potential (TI), calculated as the ratio of lethal concentration (LC_25_) to effective concentration (EC_25_) that cause 25% malformations, indicated that purified MC-LR, as well as both types of biomass, can impose a significant embryotoxic risk to freshwater vertebrates. Additional experiments with external additions of purified MC-LR to both types of biomass revealed little additive effect. The authors explained the results either by a possible decreased bioavailability of MC-LR in the complex biomass (sorption to proteins or membrane lipids) or by an overlapping effect of other unidentified metabolites present in the complex cyanobacterial bloom.

Burýšková *et al.* [157] also studied the effects of complex natural bloom samples on the African frog (*X. laevis*) embryos, but besides classical parameters (mortality, growth inhibition and malformations) evaluated in 96 h FETAX, they also assessed the effects on biochemical markers of oxidative stress and detoxification (glutathione pool, GSH; GPx activity, GR and GST activity). In general, complex biomass samples containing several genera of cyanobacteria (Fraction I) and aqueous extracts (Fraction III) were generally the most toxic fractions in terms of mortality and growth inhibition, whereas methanolic eluates passed through C18 column containing MCs (Fraction V) were less toxic. On the other hand, the same fraction (eluates) induced significant malformations in low concentrations but the effects were not related to the MCs content. Curiously, the strongest effect was caused by permeate from C18 column without detectable MCs (Fraction IV). Oedema, adverse curving of backbone, eye and mouth deformities were the most often observed malformations in cyanobacterial extracts. Also, significant growth reductions were recorded after exposure to all fractions of complex biomass, except for the eluate containing MCs. Biomarkers were affected in a variable manner and no significant or clear concentration-dependent effect was observed. Their data support the hypothesis that MCs are not the only or major toxic compounds in the complex cyanobacterial samples.

White *et al.* [158] presented the first evidence of bioaccumulation and effects of CYN-producing *C. raciborskii* in the developmental stages of an amphibian (*Bufo marinus*). Maximum average tissue concentrations of 895 and 61 µg free-CYN kg^−1^ FW were measured after exposure to live culture containing cell-bound toxin and to whole cell extracts containing extracellular toxin, respectively, which resulted in up to 66% mortality of tadpoles exposed to live cultures, whereas tadpoles exposed to whole cell extracts containing similar toxin concentrations survived. Both types of exposure lead to decreases in relative growth rate and time spent for swimming. This study, similarly to others [71,111,201,203], indicates that absorption of dissolved toxins from cell extracts is relatively low compared to the ingested cell-bound toxins.

Evidence of bioaccumulation and toxicity of cyanotoxins to other aquatic vertebrates (*i.e.*, reptiles and birds) are very scarce, and literature is mainly based on reports of mass mortality of birds and waterfowls, insufficiently linked to the presence of cyanotoxins (reviews about bird poisonings can be found in [31,85]). 

Recently, Chen *et al.* [160] reported the presence of MCs in the domestic duck *Anas platyrhynchos* and in the black-crowned night heron *Nycticorax nycticoraxs* in Lake Taihu (China). Among the various organs analyzed, they found higher amounts of MCs in the intestines (51–82 ng g^−1^ DW), liver (17–31 ng g^−1^ DW) and stomach (10–21 ng g^−1^ DW), and smaller amounts in the pancreas, gallbladder, kidney, heart, lung, spleen, gonads and muscles. They found also considerable amounts of MCs in the egg yolk (8–15 ng g^−1^ DW), which indicates a possible risk for bird embryos. In spite of this, no bird mortality was reported in this study.

One of the first experimental studies using aquatic birds reported differences between mallard ducks and ring-necked pheasants for the ANTX-a produced by the strain of *Anabaena flos-aquae* NRC-44-1 [207]. More recent experimental work has used the Japanese quail (*Coturnix coturnix japonica*) as a model bird species [208]. In this study, male Japanese quails were offered *Microcystis* biomass containing increasing doses of MCs for 10 (acute) and 30 (sub-chronic) days, but showed no mortality. However, histopathological hepatic alterations in birds were reported after exposure, including cloudy swelling of hepatocytes, vacuolar dystrophy, steatosis and hyperplasia of lymphatic centers. On a subcellular level, shrunken nuclei of hepatocytes containing ring-like nucleoli, cristolysis within mitochondria and vacuoles with pseudomyelin structures were present. Vacuolar degeneration of the testicular germinative epithelium was also found in two exposed males. Evaluation of biochemistry and haematological parameters showed increased activities of lactate dehydrogenase (LD) and a drop in the blood glucose (GLU) on day 10, only for the highest dose (46 mg MC per 10 mL^−1^ day^−1^). 

In another study with Japanese quails, Pašková *et al.* [163] studied the effects on detoxification and antioxidant enzymes as well as bioaccumulation of MCs after acute (10 days) and sub-lethal (30 days) exposure to natural cyanobacterial biomass containing MCs. Significant accumulation of MCs was observed in the liver for both test durations and slight accumulation also in the muscles of the highest treatment group from the acute test. The greatest accumulation was observed in the liver of the highest treatment group in the acute test reaching an average concentration of 43.7 ng MCs g^−1^ FW. Cyanobacterial exposure caused an increase of activity representing the activation phase of detoxification metabolism (EROD), mainly in the heart and brain tissues in the acute exposure. Also the conjugation phase of detoxification, mainly the activity GST, was altered in the liver and heart in the sub-chronic exposure. Cyanobacterial exposure also modulated oxidative stress responses including the level of glutathione and activities of glutathione-related enzymes and caused increases in lipid peroxidation in most tissues, both in acute and sub-chronic exposures. Although the Japanese quail is not representative of wild aquatic birds, they may be considered as models for future investigations of wildlife mortalities related to toxic blooms of cyanobacteria.

The only evidence of mortality of reptiles is the study of Nasri *et al.* [159], who reported a case of mortality of the freshwater terrapin turtle species *Emys orbicularis* and *Mauremys leprosa* during a *Microcystis* bloom in Lake Oubeira (Algeria, Tunisia). MC-LR, -YR and -RR were detected in this bloom extract and also in fresh carcasses of terrapin liver, viscera and muscle tissues using the GC/MS after Lemieux oxidation method and the PP2A inhibition assay. The highest level of MC-LR equivalents detected using the Lemieux oxidation-GC/MS method was found in the liver (1193 µg g^−1^ DW) and in the viscera tissues (37.2 µg g^−1^ DW) of *M. leprosa* and *E. orbicularis*, respectively, and smaller levels in the muscle (9.4–10.1 µg g^−1^ DW). The liver crumbling observed after the necropsy examination of the fresh carcasses of *M. leprosa* support the hypothesis that hepatotoxins contributed to the turtle mortalities. Although both species are prevalently carnivorous, *M. leprosa* diet is highly biased towards filamentous algae, insect larvae, earthworms, mollusks, small amphibians and tadpoles, and various aquatic plants. Thus, the most likely uptake of MCs by these turtles is from food chain. Chen *et al.* [160] also found MCs in several organs, including the liver and intestines, of the carnivorous turtle *Pelodiscus sinensis* in Lake Taihu (China), but no mortality was reported.

## 4. Exposure Mode and Experimental Conditions

The mode of exposure (living cells, extracts or purified toxin), however, is one of the main influencing factors in the toxicity of cyanobacteria. For instance, for zooplankton the effects are generally more pronounced when the animals are exposed to intact cells, even at low concentrations than when exposed to extracts or purified toxins. This basically comes from the fact that the uptake of toxins via the digestive tract is much more efficient than the dermal route; especially considering that zooplankton have a chitinous carapace. Rohrlack *et al.* [69] described the mechanism of intoxication by MCs in *Daphnia* as being primarily a digestive uptake. After digestion of cells in the midgut, where toxins accumulate, they are transported directly into the blood through the gut epithelium. These authors also showed that this transport involves the loss of cell-to-cell contact in the midgut epithelium, enhancing the permeability to MCs.

Few studies have made use of pure toxins, primarily due to the high cost and high dosage needed for these chemicals. DeMott *et al.* [71] found values of LC_50_ (48 h) for MC-LR and NOD varying from 9.6 to 21.4 mg MC-LR L^−1^ and from 3.9 to 14.1 mg NOD L^−1^, respectively for several *Daphnia* species, and from 0.45 to 1.0 mg MC-LR L^−1^ and 0.52 to 1.25 mg NOD L^−1^ for *Diaptomus birgei*, indicating that copepods seem to be more sensitive to both hepatotoxins than cladocerans. Yasuno and Sugaya [209] found LC_50_ (48 h) values for different MC variants (YR + LR and RR) ranging from 1.0 to 2.3 mg L^−1^ for *Moina macrocopa*, suggesting that this cladoceran is even more sensitive to MCs than *Daphnia*. Gilbert [154] found LC_50_ (48 h) values for ANTX-a varying from 0.18 to 4.39 mg L^−1^ for different rotifer species, indicating that these organisms seem to be very sensitive to this toxin. On the other hand, Reinikainen *et al.* [111] did not find a significant negative effect of ANTX-a and NOD on the survivorship of the copepods *Eurytemora affinis* and *Acartia bifilosa* as well as in the frequency of egg hatching of *E. affinis*, but found a LC_50_ (48 h) of 0.27 mg L^−1^ for this copepod submitted to MC-LR.

Despite the fact that the use of purified toxins may provide direct evidence of the effects of cyanotoxins on zooplankton, the concentrations employed are relatively high and not ecologicaly relevant [71,111], rarely occurring in nature, even in heavy cyanobacterial blooms. Notwithstanding calanoid copepods may show efficient uptake of dissolved NOD [93], this may not be valid for other toxins and organisms [71,111,201,203,210,211,212]. Because cyanotoxins are mostly endotoxins, released out of the cells only during bloom senescence and relatively fast degraded in the water, the concentration of dissolved toxins is relatively low in the aquatic environment [213]. Therefore, it is likely that aquatic organisms are exposed to low levels of dissolved toxins in the natural environment and thus direct uptake of dissolved toxins from the medium does not represent the typical and ecologically relevant exposure route of aquatic organisms, that is via the food web [12,39,40]. 

Nevertheless, many studies found stronger effects of extracts than purified toxins. According to some authors [156,157,203], this could be due to synergistic actions of different toxins in complex matrixes of bloom samples increasing toxicity considerably. It has been also suggested that other bioactive substances such as lipopolysaccharides (LPS), mueggelone and lupenyl acetate [203] could be responsible for the higher toxicity of extracts. Thus, care must be taken when trying to correlate results with purified toxins to eithet blooms or cell cultures since many other coumpouds may be present and interact in these complex matrix samples.

The presence and concentration of alternative (*i.e.*, nutritive) food is another factor that influences the experimental results. The operational concept of cyanobacterial toxicity generally involves the comparison of treatments with single diets of cyanobacteria with a treatment without any source of food (*i.e.*, starvation) [214]. It has been assumed that if animals die faster in the treatments with cyanobacteria than under starvation, then cyanobacteria are toxic. However, equivocal interpretations may arise due to large differences among species regarding resistance to starvation or to toxic cyanobacteria and their toxins [72]. Also, survivorship can be significantly improved if alternative food is added to treatments with cyanobacteria [72,151,215,216,217,218].

Other factors such as temperature [219,220,221], genotype [218,222,223,224] and presence of predators [224] have been shown to affect the response of zooplanktonic organisms to toxic cyanobacteria.

The exposure route has been considered of great importance in determining the effects of cyanotoxins on fish, as dissolved toxins cause much less harmful effects or no mortality at all, when compared to the same doses applied orally. Although in natural conditions the uptake of toxins is mainly through the ingestion of cells and absorption in the gastro-intestinal tract (via bile acid system), the uptake can occur, though in lower proportion, through the gills and epidermis [210]. In some experimental studies, however, gastro-intestinal exposure to cyanotoxins is made in a rather artificial way, introducing cyanobacterial extracts directly in the stomach of fish using tubes (*i.e.*, gavage), or using mixtures of cell extracts and agar-agar in the form of a gelatin offered *ad libitum* [225,226,227,228]. Others have applied intraperitonial injections of lised cell concentrates from lab cultures or natural bloom samples [225,227,229]. Although these techniques are useful in the study of the toxicokinetics and histopathological alterations, they do not represent de mode of exposure in the natural environment. Magalhães *et al.* [170], however, found large mass of toxic cells in the stomach of tilapia (*Tilapia rendalli*) from Jacarepaguá Lagoon (RJ, Brazil), and a high content of MCs in the vicera, liver and muscle, which demonstrate the ingestion and assimilation of these toxins in natural conditions. Also, Zhang *et al.* [230] measured high concentrations of MCs in the intestine of carp (*H. molitrix*), but did not detect any toxin in the adipose tissue, suggesting that oral ingestion is the most likely exposure route. Therefore, omnivorous and planktivorous fish species, such as tilapia and carp, are most likely exposed to cyanotoxins through oral consumption.

## 5. Tolerance/Resistance to Cyanotoxins

Cyanobacterial blooms are short-lived in temperate regions, whereas they tend to persist througout the year, sometimes for several years in the tropics. This has lead to the hypothesis that tropical zooplankton might have developed higher tolerance/resistance to toxic cyanobacteria and their toxins. In general, tropical cladoceran species have smaller adult body sizes, shorter life spam, reach maturity earlier and have higher rate of population increase than their temperate counterparts [231]. Thus, it is likely that small, fast-growing species that invest heavily in reproduction at early life stages are more sensitive to food limitation (and thus to cyanobacteria) than species that allocate more energy in reaching a larger size in the pre-reproductive phase and are less succeptible to food limitation [72,232]. Ferrão-Filho *et al.* [72] showed, however, that tolerance is not necessarily linked to geographical origin. Tropical *Ceriodaphnia cornuta* had much higher tolerance to toxic *Microcystis* than other cladocerans, including the temperate *Daphnia pulex*, *D. pulicaria* and *D. similis*. Tropical *Moinodaphnia macleayi* (*i.e.*, *Moina minuta*) was, however, the most sensitive species among the cladocerans tested. The authors concluded that sensitive and resistant species occur in both geographic regions and suggested that differences are much more related to the life history and energy allocation strategy of the species. 

High intraespecific (clonal) variation in the sensitivity to toxic cyanobacteria has been observed [216,217,221,222,223]. For instance, *D. pulicaria* clones isolated from eutrophic lakes have been found to be more tolerant to diets of toxic *Microcystis* than clones from oligotrophic ones [233]. Selection of resistant clones, from parthenogenetic organisms that have sexual reproduction events has been pointed out as a factor responsible for the appearance of resistance to toxic cyanobacteria in lakes that passed through eutrophication process along decades and became dominated by cyanobacteria [234,235]. Hairston *et al.* [234] showed that clones of *D. galeata*, originated from resting eggs taken from the sediment of Lake Constance (Europe), with ages before (1962–1971), during (1978–1980) and after (1992–1997) peak eutrophication, presented differential sensitivity to toxic *Microcystis*. Clones with ages before eutrophication were significantly more sensitive (lower LC_50_) than clones with ages after peak eutrophication. Using data from the same *Daphnia* clones and strain of *Microcystis*, Hairston *et al.* [235] showed that this increased resistance is expressed also in the growth potential of the species (measured as juvenile growth rate, *g_j_*). Clones from peak eutrophication had a significant increase in the overall fitness, expressed as a reduction in the slope of the mean reaction norm of log(*g_j_*) over time, suggesting that phenotypic plasticity evolved over the period studied. They concluded that increased cyanobacterial densities acted on the *Daphnia* population as an important selection agent to which the *Daphnia* population responded, and that these selection responses are strikingly rapid, occurring within the time spam of little more than a decade.

The development of tolerance in cladocerans pre-exposed to toxic cyanobacteria has been reported in some studies [63,64,65]. Maternal pre-exposure to toxic *Microcystis* indeed may result in enhanced tolerance to MCs by the following generations. Gustafsson *et al.* [64] showed that females pre-exposed to a toxic strain of *M. aeruginosa* had three generations (F1, F2 and F3) of descendents that presented an improvement in their fitness (intrinsic rate of population increase, *r*), suggesting that the development of tolerance to toxic cyanobacteria is a inducible defense and can be transferred from mother to offspring. The authors argued that this may reflect an induction response of the detoxication mechanism to the continuous exposure to toxic cyanobacteria and that such phenotypic plasticity could be an important adaptation for clonal animals to withstand rapid variations in toxin concentrations in the environment.

The behavioral resistance has also been pointed out as a mechanism that confers some resistance to the presence of toxic cyanobacteria [60,236]. DeMott and Moxter [60] showed that copepods are more selective, avoiding the ingestion of toxic cyanobacteria, but that selectivity depends upon prey availability and on degree of starvation (*i.e.*, “hunger”). DeMott [236] also found that different *Daphnia* species presented variable resistance to the ingestion of toxic cyanobacteria. Some species showed continued low feeding rate after 24 h exposure, showing high resistance to the ingestion of toxic cells, while *D. magna* showed a strong inhibition but recovered feeding rates to control levels after 24 h. The authors argued that recovery pattern shown by some species after 24 h acclimatization is consistent with an important role for behavioral flexibility in controlling the feeding rate. Therefore, “hunger” is suggested as an important factor modulating behavioral resistance to toxic cyanobacteria. Although cladocerans, especially of the genus *Daphnia*, have been considered generalist feeders, incapable to discriminate food particles by nutritive value and toxicity as do copepods [60], video recording and computerized image analysis have showed that *D. pulicaria* is able to discriminate between *Microcystis* and *Scenedesmus* [108]. Animals exposed to toxic cells of *Microcystis* showed decreased mandibular movements and decreased appendages beating rates, while showing increased labral rejection rate. These effects were totally reversible when animals were exposed back to control medium, suggesting a behavioral response rather than intoxication. When purified MC-LR was added, however, there was no recovery in any of these parameters, indicating a toxigenic response [108]. 

Despite the intra- and interespecific variability in the sensitivity to toxic cyanobacteria, evidence of physiological tolerance to cyanotoxins has not been consistently demonstrated. One study [40], however, gave strong evidence that some *Daphnia* clones are not affected by the consumption of MC-LR containing food. They tested this hypothesis by adding pure MC-LR to freeze-dried good food (*Chlorella*) and feeding two *Daphnia* clones with different sensitivity to toxic *Microcystis*. Surprisingly, the *Daphnia* that performed better on a diet containing live *Microcystis* showed reduced population growth when exposed to MC-LR-treated *Chlorella*, whereas the *Daphnia* that performed poorly on the diet containing live *Microcystis* was not affected by the diet containing MC-LR. Although it was not clear why there was this inverse response, the authors argued that exposure to toxin-producing cyanobacteria or pure cyanotoxins, may upregulate greater resistance to the effects of these foods, so it is possible that the resistant clones can upregulate resistance when exposed to “cues” by the live cyanobacteria, but not when cued only by pure MC-LR. Both this and the study of Ghadouani *et al.* [108], suggest that compounds other than MCs may act as “chemical cues”, being responsible for the behavioral avoidance (*i.e.*, decreased ingestion) of toxic cells, which can confer some resistance to sensitive species.

Remarkable differences in the sensitivity of different *Daphnia* species to the same toxic strain of cyanobacteria have been observed and are probably related to physiological tolerance to cyanotoxins. Ferrão-Filho *et al.* [10] showed that while *D. pulex* was very sensitive to a STXs-producer strain of *C. raciborskii* (T3), being immobilized at concentrations as low as 100 cells mL^−1^ (~8.5 pg STX equivalents L^−1^), its tropical counterpart *D. gessneri* did not suffer any effect even at much higher concentrations (10^4^ cells mL^−1^ or ~9370 pg STX equivalents L^−1^). A tropical clone of *Moina micrura* was also sensitive to *C. raciborskii*, but at intermediate concentrations (10^3^ cells mL^−1^ ~ 937 pg STX equivalents L^−1^). Those cladocerans were, however, not affected at all by a non STXs-producing strain of *C. raciborskii*, strongly suggesting that the effect was caused by cell-bound STXs. Ferrão-Filho *et al.* [153] showed that a *D. pulex* acute toxicity bioassay can be even used as a detection toll for STXs in raw water, given its high sensitivity and fast response. Acute and chronic toxicity data (non published results) show, however, that other tropical cladoceran species (*i.e.*, *Diaphanosoma birgei* and *Ceriodaphnia cornuta*) are not sensitive to STXs-producer *C. raciborskii*, being even able to grow and and reproduce in diets with this cyanobacterium. However, whether these species have a behavioral resistance (e.g., avoidance, rejection) or physiological tolerance to these saxitoxin-producer strains remains unclear.

Differences in the sensitivity of organisms to toxins can be explained by differences in the activity of detoxication enzymes, such as those of the cytochrome P-450 complex and GSTs [35,194,237]. The GSTs are part of the enzymes of Phase II detoxifying system, developing an important role in the initiation of detoxication process and being present in all taxonomic groups [238]. The conjugation reaction of the sulphydril group of glutathione (GSH) with the eletrophyllic group of xenobiotic compounds and toxins, catalyzed by GST, makes the reaction products less toxic and more hydrosoluble, facilitating the excretion [35]. Some studies pointed out an increase in the activity of these enzymes, as well as in the formation of conjugates between GSH and MCs, as responsible by the reduction of the toxicity of these toxins in aquatic organisms [107,115,119,141,147,194,195]. Therefore, the detoxication process via GST, well known for xenobiotics, can be utilized by several taxa of aquatic organisms to survive under stress caused by cyanobacteria [194]. 

Intra-specific variation in resistance to cyanotoxins has also been related to genetic adaptation. Bricelj *et al.* [239,240] found evidence of selection for resistance to paralytic shellfish toxins (PST) during the early life history of soft-shell clam (*Mya arenaria*) populations related to a natural mutation of a single amino acid residue. This mutation causes a 1000-fold decrease in affinity at the saxitoxin-binding site in the sodium channel pore of resistant clams. Thus, it is likely that this local, genetic adaptation may occur also in freshwater habitats, leading to an increase in resistance and bioaccumulation potential of cyanotoxins in aquatic animals, further increasing the risk of human contamination.

## 6. Conclusions

The high variability found among the different bioaccumulation studies made inter-comparison difficult, mainly due to the use of different methodologies in the analysis of toxins, to different exposure modes and source of toxins (*i.e.*, live cells, extracts or purified toxins). Also, the different ingestion, digestion and detoxification capabilities of the different taxa are factors that might have increased viariablility. 

Although bioaccumulation and transferring of cyanotoxins do occur, biodilution of MCs appears to be the dominant process based on the low BMF found for several aquatic consumers [86]. This is also suggested by the higher zooplankton MCs burden in relation to planktivorous fish and the lower toxin burden in carnivores compared to other fish trophic guilds (but see [86]). Low hydrophobicity, especially of MC-LR (e.g., [241]) and detoxication of MCs [3,35,194] are factors that may contribute to biodilution, preventing biomagnification to takes place. Nevertheless, biomagnification of MCs and other toxins may occur [75,76,77,78,140,143,144] and should not be negleted. 

Even if cyanotoxins may adversely affect aquatic organisms, their ecological role is still poorly understood. The “chemical defense” hypothesis has been consistently questioned simply because not a single study (but see [40]) has so far unequivocally shown that the adverse effect is linked to the presence of a specific, cell-bound known toxin. Morphological and nutritional factors are, in most cases, difficult to disentangle from toxic effects, causing confounding synergistic effects. Also, the same strain can produce more than one toxic metabolite, which makes it difficult to characterize a cause-effect relationship. Experiments with pure toxins, which might provide direct evidence of the deleterious effects of cyanotoxins are, on the other hand, rarely performed in realistic exposure-concentration conditions. Besides that, this exposure route does not represent the most ecologically relevant one, *i.e.*, through direct consumption of toxic cyanobacteria or, indirectly, through the food web. Therefore, future studies should focus in the planktonic trophic relationships and in the food web-mediated transfer and effects of toxic cyanobacteria.

The majority of studies in freshwater systems have focused on MCs and much less research has been dedicated to the other toxins. This is probably due to the fact that not only MCs-producer cyanobacteria dominate in most freshwater ecosystems but also to the better knowledge of their toxicity and mechanism of action. Nevertheles, many studies have shown no effect of purified MCs or sample fractions containing MCs, but higher effects of crude extracts from both cultured and natural biomass. Thus, compounds other than MCs may be responsible for some of the effects observed on aquatic organisms. Further studies of chemical isolation and characterization of new compounds with biological activity are, therefore, greatly required. Also, special attention should be payed to STXs and CYN both produced by *Cylindrospermopsis*, a genus in plentiful expansion worldwide, and to BMAA potentially produced by all cyanobacteria.

Given the number of contradictory results regarding the effects of cyanotoxins in biomarker studies, especially on the enzymes of the detoxication system, more studies of the effects cyanotoxins at the biochemical level should be performed to the better understanding of the mechanism of action of these toxins in aquatic organisms. On the other hand, oxidative stress seems to be an important mechanism by which cyanotoxins can be harmful to aquatic organisms.

Finally, studies about the effects of cyanobacteria and bioacummulation of their toxins in the tropical region are of great importance, as the incidence and persistence of toxic blooms are greater than in temperate regions. As bioaccumulation of toxins are expected to be higher in the tropics and may reach upper trophic levels, human health might be at higher risk, especially considering that most undeveloped countries are situated in these latitudes and that the control of toxins in food and water supply is not as well regulated. 

## Supplementary Files

Supplementary File 1: PDF-Document (PDF, 232 KB)
