# Supplementary material for: Cyanotoxins: Bioaccumulation and Effects on Aquatic Animals
Source: Mar Drugs. 2011 Dec 16;9(12):2729–72. doi: 10.3390/md9122729 (PMC3280578; doi:10.3390/md9122729)
Supplement: Supplementary File 1: — PDF-Document (PDF, 232 KB) [file marinedrugs-09-02729-s001.pdf]

Supplementary Information

Table S1. Cyanotoxins content in aquatic invertebrates. Concentrations of microcystins are given in equivalents of MC-LR, and concentrations of saxitoxins are given in equivalents of STX. Concentrations represent the range for the different organs/tissues in [(a)] µg g^{-1} DW, [(b)] ng individual^{-1}, [(c)] µg gC^{-1} or [(d)] pmol individual^{-1}. Concentrations in µg g^{-1} WW were converted to µg g^{-1} DW multiplying by 10. MC = microcystins; NOD = nodularin; CYN = cylindrosdpermopsin; STX = saxitoxins; ANTX-a = anatoxin-a; BMAA = β-*N*-methylamino-L-alanine. I-EC = Ion-Exchange Chromatography; HPLC = High Performance Liquid Chromatography; LC-MS = Liquid Chromatography–Mass Spectrometry; ELISA = Enzyme Linked Immunosorbant Assay; PP2A = Phosphatase 2A Inhibition Assay; PP1 = Phosphatase 1 Inhibition Assay; MMPB = 3-metoxi-2methyl-4-phenylbutiric acid; EC-MS = Capillary Eletrophoresis–Mass Spectrometry; GC-MS = Gas Chromatography–Mass Spectrometry; DW = dry weight; TEH = Total Extractable Hepatotoxins; ND = not detectable.

Group of organism/Taxa	Dominant cyanobacteria	Toxin	Analytical method	Concentration	Reference
**ZOOPLANKTON**					
Natural community	*Microcystis* spp.	MC-LR, -RR, -YR	I-EC	75.0–1387 (a)	Watanabe *et al.* (1992)[1]
Natural community	*M. aeruginosa*	MC-LR	HPLC/PP2A	ND–67.0 (a)	Kotak *et al.* (1996)[2]
Daphnia magna (Cladocera)—Lab experiment	*M. aeruginosa*	MC	ELISA	0.2–24.5 (a)	Thorstrup and Christoffersen (1999)[3]
Daphnia magna (Cladocera)—Field experiment	*M. aeruginosa*	MC	ELISA	0.3–15.5 (a)	
D. parvula (Cladocera)	*M. aeruginosa*, *Chroococcus minor*	MC	ELISA	ND–71.2 (b)	Mohamed (2001)[4]
Natural community	*M. aeruginosa*	MC	ELISA	0.3–16.4 (a)	Ferrão-Filho *et al.* (2002)[5]
Eurytemora affinis (Copepoda)	*Nodularia spumigena*	NOD	ELISA/PP1	0.031–0.101 (b)	Lehtiniemi *et al.* (2002)[6]
Acartia bifilosa (Copepoda)	*N. spumigena*	NOD	ELISA/PP1	0.011–0.013 (b)	Kozlowsky-Suzuki *et al.* (2003)[7]
Eurytemora affinis (Copepoda)	*N. spumigena*	NOD	ELISA/PP1	0.011–0.024 (b)	
Acartia tonsa (Copepoda)	*N. spumigena*	NOD	Radiolabeled NOD	0.37 (c)	Karjalainen *et al.* (2003)[8]
Eurytemora affinis (Copepoda)	*N. spumigena*	NOD	Radiolabeled NOD	0.60 (c)	
Strobidium sulcatus (Ciliata)	*N. spumigena*	NOD	Radiolabeled NOD	1.55 (c)	
D. magna (Cladocera)	*Cylindrospermopsis raciborskii*	CYN	HPLC	0.020–0.025 (b)	Nogueira *et al.* (2004)[9]
D. magna (Cladocera)	*Aphanizomenon issatschenkoi*	STXs	HPLC	0.07–0.38 (d)	Nogueira *et al.* (2004)[10]
Natural community	*M. aeruginosa*, *Planktothrix agadhii*	MC	LC-MS/HPLC/ELISA	ND–1226 (a)	Ibelings *et al.* (2005)[11]
Natural community	*M. aeruginosa*	MC	PP2A/HPLC	0.7–1.02 (a)	Lehman *et al.* (2005)[12]
Several species	*N. spumigena*	NOD	ELISA	ND–6.2 (a)	Karjalainen *et al.* (2006)[13]

Table S1. *Cont.*

Eurytemora affinis (Copepoda)	*N. spumigena*	NOD	ELISA/PP1	12.1–46.4 (a)	
Natural community	*Microcystis* sp.	MC	ELISA	5.0–25.0 (a)	Smith and Haney (2006)[14]
D. pulicaria (Cladocera)	*Planktothrix agardhii*, *P. rubescens*	MC	PP2A	74.0–1099 (a)	Oberhaus *et al.* (2007)[15]
Natural community	*Microcystis* sp., *C. raciborskii*, *Anabaena circinalis*	MC	ELISA	5.0–63.2 (a)	Rocha (2007)[16]
Pleopsis polyphemoides (Cladocera)	*N. spumigena*	NOD	ELISA	ND–23.6 (a)	Karjalainen *et al.* (2008)[17]
Natural community (*E. affinis* and *Pseudodiaptomus forbesii*)	*Microcystis* sp.	MC	PP2A/HPLC	0.18–5.93 (a)	Lehman *et al.* (2008)[18]
Amphipod species	*Microcystis* sp.	MC	PP2A/HPLC	0.74–5.18 (a)	
Worms (unknown species)	*Microcystis* sp.	MC	PP2A/HPLC	12.0 (a)	
Jellyfish (unknown species)	*Microcystis* sp.	MC	PP2A/HPLC	2.2 (a)	
Eurytemora affinis (Copepoda)	*N. spumigena*	NOD	ELISA	0.04–0.76 (b)	Gorokhova and Engström-Öst (2009)[19]
Eurytemora affinis (Copepoda)	*N. spumigena*	NOD	HPLC-MS/LC-ESI-MS	0.0013–0.0143 (b)	Sopanen *et al.* (2009)[20]
Natural community	*Microcystis* sp.	MC	PP2A/HPLC	0.40–1.43 (a)	Lehman *et al.* (2010)[21]
Amphipod species	*Microcystis* sp.	MC	PP2A/HPLC	0.77 (a)	
**CRUSTACEANS**					
Cherax quadricarinatus (Decapoda)	*C. raciborskii*	CYN	HPLC	0.54–4.3 (a)	Saker and Eaglesham (1999)[22]
Echinogammarus ischnus (Amphipoda)—Field data	*M. aeruginosa*	MC	ELISA	0.2 (a)	Babcock-Jackson (2000)[23]
Echinogammarus ischnus (Amphipoda)—Lab experiment	*M. aeruginosa*	MC	ELISA	0.2–0.7 (a)	
Procambarus clarkii (Decapoda)	*M. aeruginosa*	MC	ELISA	0.4–2.9 (a)	Vasconcelos *et al.* (2001)[24]
Mysis relicta (Decapoda)	*N. spumigena*	NOD	ELISA/PP1	0.5–0.74 (a)	Engström-Öst *et al.* (2002)[25]
Crab—unidentified species (Decapoda)	Picoplanktonic cyanobacteria (unidentified)	MC	ELISA	ND–1.0 (a)	Magalhães *et al.* (2003)[26]
Shrimp—unidentified species (Decapoda)	Picoplanktonic cyanobacteria (unidentified)	MC	ELISA	ND–0.09 (a)	
Palaemon modestus (Decapoda)	*Microcystis* spp., *Anabaena* sp.	MC	LC-MS	ND–9.8 (a)	Chen and Xie (2005)[27]
Procambarus clarkii (Decapoda)	*Microcystis* spp., *Anabaena* sp.	MC	LC-MS	0.05–10.0 (a)	

Table S1. *Cont.*

Macrobrachium nipponensis (Decapoda)	*Microcystis* spp., *Anabaena* sp.	MC	LC-MS	ND–12.4 [(a)]	
Panaeus monodon (Decapoda)	*Oscillatoria* sp., *Pseudoanabaena* sp., *Microcystis* spp., *Aphanocapsa* sp., *Romeria* sp.	MC/NOD (TEH)	LC-MS	0.006–0.08 [(a)]	Kankaanpää *et al.* (2005)[28]
Neomysis integer (Decapoda)	*N. spumigena*	NOD	Radiolabeled NOD	0.77 [(a)]	Karjalainen *et al.* (2005)[29]
Gammarus zaddachi (Amphipoda)	*N. spumigena*	NOD	ELISA	ND–0.002 [(c)]	Korpinen *et al.* (2006)[30]
Chasmagnathus granulatus (Decapoda)	*M. aeruginosa*	MC	ELISA	0.02–0.32 [(a)]	Dewes *et al.* (2006)[31]
Neohelice granulata (Decapoda)	*Microcystis* sp.	MC	ELISA	0.3–0.34 [(a)]	Dewes *et al.* (2007)[32]
Macrobrachium nipponensis (Decapoda)	*Microcystis* spp.	MC	ELISA/LC-MS	ND–0.76 [(a)]	Song *et al.* (2007)[33]
Macrobrachium nipponensis (Decapoda)	*M. aeruginosa*	MC-LR, -LR-GSH, -LR-Cys	LC-MS	up to 4.9 [(a)]	Zhang *et al.* (2009)[34]
Panaeus duorarum (Decapoda)	Undetermined cyanobacterial blooms	BMAA	HPLC-LC/MS/MS	55–3042	Brand *et al.* (2010)[35]
Callinectes sapidus (Decapoda)	Undetermined cyanobacterial blooms	BMAA	HPLC-LC/MS/MS	ND–6976	
Callinectes sapidus (Decapoda)	*Microcystis* sp., *Anabaena* spp.	MC	ELISA	ND–8.20 [(a)]	Garcia *et al.* (2010)[36]
**MOLLUSKS**					
Ellipio campanatus, *Corbicula fluminea* (Bivalvia)	*Aphanizomenon flos-aquae*	STXs	HPLC	0.4–0.6 [(a)]	Sasner *et al.* (1984)[37]
Anodonta cygnea (Bivalvia)	*Oscillatoria agardhii*	MC	HPLC	70–130 [(a)]	Eriksson *et al.* (1989)[38]
Anodonta cygnea (Bivalvia)	*Oscillatoria agardhii*	MC	HPLC	30.0 [(a)]	Lindholm *et al.* (1989)[39]
Mussel—unidentified species (Bivalvia)	Unspecified	MC	CE-MS	6.0 [(a)]	Chen *et al.* (1993)[40]
Alanthyria condola (Bivalvia)	*Anabaena circinalis*	STXs	HPLC	0.8–6.2 [(a)]	Negri and Jones (1995)[41]
Mytilus galloprocincialis (Bivalvia)	*M. aeruginosa*	MC	HPLC	1.0–10.5 [(a)]	Vasconcelos (1995)[42]
Helisoma trivolvis (Gastropoda)	*M. aeruginosa*, *A. flos-aquae*,	MC	HPLC	ND–11.0 [(a)]	Kotak *et al.* (1996)[2]
Lymnaea stagnalis (Gastropoda)	*Gomphosphaeria* sp.	MC	HPLC	ND–96.0 [(a)]	
Physa gyrina (Gastropoda)	*Anabaena flos-aquae*	MC	HPLC	ND–121 [(a)]	
Anodonta grandis simpsoniana (Bivalvia)	*M. aeruginosa*, *Anabaena* spp., *Aphanizomenon flos-aquae*	MC	PP2A	0.02–1.35 [(a)]	Prepas *et al.* (1997)[43]
Unio douglasiae (Bivalvia)	*M. aeruginosa*	MC	HPLC-MS	12.0–27.0 [(a)]	Watanabe *et al.* (1997)[44]
Anodonta woodiana (Bivalvia)	*M. aeruginosa*	MC	HPLC-MS	2.1 [(a)]	

Table S1. *Cont.*

Mytilus edulis—Lab experiment (Bivalvia)	*M. aeruginosa*	MC	Limieux-GC-MS	3369 (a)	Williams *et al.* (1997)[45]
Mytilus edulis—Lab experiment (Bivalvia)	*M. aeruginosa*	MC	PP2A	2.0 (a)	
Mytilus edulis—Field sample (Bivalvia)	*M. aeruginosa*	MC	Limieux-GC-MS	ND–634 (a)	
Mytilus edulis—Field sample (Bivalvia)	*M. aeruginosa*	MC	PP2A	0.17–0.22 (a)	
Mytilus galloprocincialis (Bivalvia)	*M. aeruginosa*	MC	HPLC-MS	2.0–16.0 (a)	Amorim and Vasconcelos (1999)[46]
Helisoma trivolvis (Gastropoda)	*Microcystis* spp.	MC	HPLC	ND–40 (a)	Zurawell *et al.* (1999)[47]
Lymnaea stagnalis (Gastropoda)	*Microcystis* spp.	MC	HPLC	ND–140 (a)	
Physa gyrina (Gastropoda)	*Microcystis* spp.	MC	HPLC	ND–129 (a)	
Dreissena polymorpha (Bivalvia)—Field data	*M. aeruginosa*	MC	ELISA	0.213 (a)	Babcock-Jackson (2000)[23]
Dreissena polymorpha (Bivalvia)—Lab experiment	*M. aeruginosa*	MC	ELISA	up to 0.203 (a)	
Mytilus edulis (Bivalvia)	*Aphanizomenon* sp., *N. spumigena*, *Anabaena* sp.	NOD/MC	ELISA	ND–2.15(a)	Sipiä *et al.* (2001)[48]
Mytilus edulis (Bivalvia)	*Aphanizomenon flos-aquae*, *N. spumigena*	NOD/MC	ELISA/ MALDI-TOF-MS	0.040–1.49(a)	Sipiä *et al.* (2002)[49]
Macoma balthica (Bivalvia)	*Aphanizomenon flos-aquae*, *N. spumigena*	NOD/MC	ELISA	0.10–0.13(a)	
Anodonta woodiana (Bivalvia)	*Microcystis* spp.	MC	HPLC	ND–12.6 (a)	Yokoyama and Park (2002)[50]
Cristaria plicata (Bivalvia)	*Microcystis* spp.	MC	HPLC	ND–297 (a)	
Unio douglasiae (Bivalvia)	*Microcystis* spp.	MC	HPLC	ND–420 (a)	
Macoma balthica (Bivalvia)	*N. spumigena*	TEH/NOD-like compound	ELISA/HPLC/ MALDI-TOF-MS	0.16–30.3 (a)	Lehtonen *et al.* (2003)[51]
Sinotaia histrica—Field sample (Gastropoda)	*M. aeruginosa*, *Anabaena spiroides*	MC	HPLC	ND–19.5 (a)	Ozawa *et al.* (2003)[52]
S. histrica—Lab experiment (Gastropoda)	*M. aeruginosa*	MC	HPLC	ND–436 (a)	
Unio douglasiae (Bivalvia)—15 °C	*Microcystis ichytioblade*	MC	HPLC	ND–200 (a)	Yokoyama and Park (2003)[53]
Unio douglasiae (Bivalvia)—25 °C	*Microcystis ichytioblade*	MC	HPLC	0–630 (a)	
Dreissena polymorpha (Bivalvia)	*M. aeruginosa*	MC	LC-MS/MMPB	9.1–16.3 (a)	Dionisio-Pires *et al.* (2004)[54]
Pinctada máxima (Bivalvia)	*Trichodesmium erythraeum*	STX	HPLC	0.73 (a)	Negri *et al.* (2004)[55]

Table S1. *Cont.*

Anodonta cygnea (Bivalvia)	*A. issatschenkoi*	STXs	HPLC	0.26 [(a)]	Pereira *et al.* (2004)[56]
Anodonta cygnea (Bivalvia)	*C. raciborskii*	CYN	HPLC	2.9–61.5 [(a)]	Saker *et al.* (2004)[57]
Anodonta woodiana (Bivalvia)	*Microcystis* spp.	MC-LR,-RR,-YR	LC-MS/HPLC	0.007–7.88 [(a)]	Chen and Xie (2005)[58]
Hyriopsis cumingii (Bivalvia)	*Microcystis* spp.	MC-LR,-RR,-YR	LC-MS/HPLC	0.015–20.65 [(a)]	
Cristaria plicata (Bivalvia)	*Microcystis* spp.	MC-LR,-RR,-YR	LC-MS/HPLC	0.017–38.48 [(a)]	
Lamprotula leai (Bivalvia)	*Microcystis* spp.	MC-LR,-RR,-YR	LC-MS/HPLC	0.004–13.23 [(a)]	
Bellamya aeruginosa (Gastropoda)	*Microcystis* spp., *Anabaena* sp.	MC	LC-MS	ND–7.42 [(a)]	Chen *et al.* (2005)[59]
L. stagnalis—Juvenile (Gastropoda)	---	MC-LR	ELISA	0.068 [(a)]	Gérard *et al.* (2005)[60]
L. stagnalis—Adult (Gastropoda)	---	MC-LR	ELISA	0.014 [(a)]	
Dreissena polymorpha (Bivalvia)	*M. aeruginosa*, *Planktothrix agardhii*	MC	LC-MS/HPLC/ ELISA	ND–30.0 [(a)]	Ibelings *et al.* (2005)[11]
Clams (unknown species)	*M. aeruginosa*	MC	PP2A/HPLC	0.02 [(a)]	Lehman *et al.* (2005)[12]
Mytilus edulis (Bivalvia)	*N. spumigena*	NOD	LC-ESI-MS	ND–244.9 [(a)]	Svensen *et al.* (2005)[61]
Mytilus edulis (Bivalvia)	*N. spumigena*	NOD	LC-ESI-MS	0.03–13.75 [(a)]	Strogyloudi *et al.* (2006)[62]
Hydrella menziesi (Bivalvia)	*Anabaena* spp., *Microcystis* spp.	MC	LC-MS/ELISA	ND–0.97 [(a)]	Wood *et al.* (2006)[63]
Melanoides tuberculata (Gastropoda)	*C. raciborskii*	CYN	HPLC	ND–250 [(a)]	White *et al.* (2006)[64]
Anodonta sp.—Lake Kastoria (Bivalvia)	*M. aeruginosa*, *M. novacekii*	MC	PP2A	3.27 [(a)]	Gkelis *et al.* (2006)[65]
Viviparus contectus—Lake Pamvotis (Bivalvia)	*M. aeruginosa; A. flos-aquae*	MC	PP2A/ELISA	1.66–3.50 [(a)]	
Lymnaea stagnalis—Juvenile (Gastropoda)	*P. agardhii*	MC	ELISA	ND–194.8 [(a)]	Lance *et al.* (2006)[66]
Lymnaea stagnalis—Adults (Gastropoda)	*P. agardhii*	MC	ELISA	ND–135.8 [(a)]	
Cristaria plicata (Bivalvia)	*Microcystis* spp.	MC-LR, -RR, -YR	LC-MS	0.004–24.69 [(a)]	Chen and Xie (2007)[67]
Hyriopsis cumingii (Bivalvia)	*Microcystis* spp.	MC-LR, -RR, -YR	LC-MS	0.005–13.64 [(a)]	
Lamprotula leai (Bivalvia)	*Microcystis* spp.	MC-LR, -RR, -YR	LC-MS	0.004–7.37 [(a)]	
Mytilus edulis (Bivalvia)	*N. spumigena*	NOD	LC-MS	up to 1.1 [(a)] 0.6–9.8 [(b)]	Kankaanpää *et al.* (2007)[68]
Mytilus edulis (Bivalvia)	*N. spumigena*	NOD	ELISA	0.003–0.14 [(a)]	Mazur-Marzec *et al.* (2007)[69]
Cristaria plicata (Bivalvia)	*Microcystis* spp.	MC	ELISA/LC-MS	0.045–0.73 [(a)]	Song *et al.* (2007)[33]
Bellamya aeruginosa (Gastropoda)	*Microcystis* spp.	MC	ELISA/LC-MS	0.0656–2.34 [(a)]	
Sinotaia histrica (Gastropoda)	*Microcystis* spp., *A. flos-aquae*	MC	HPLC	0.07–23.2 [(a)]	Xie *et al.* (2007)[70]

Table S1. *Cont.*

Potamopyrgus antipodarum (Gastropoda)	*P. agardhii*	MC	ELISA	ND–4.9 [(a)]	Lance *et al.* (2008)[71]
Clams (unknown species)	*Microcystis* sp.	MC	PP2A/HPLC	0.15–0.37 [(a)]	Lehman *et al.* (2008)[18]
Mytilus edulis (Bivalvia)	*N. spumigena*	NOD	LC-MS	0.012–0.08	Sipiä *et al.* (2008)[72]
Mytilus galloprocincialis (Bivalvia)	*M. aeruginosa*	MC	ELISA	ND–0.38 [(a)]	Fernandes *et al.* (2009)[73]
Sphaerium corneum (Bivalvia)	*M. viridis*, *P. agardhii*	MC	ELISA	ND–0.04 [(a)]	Gérard *et al.* (2009)[74]
Pisidum sp. (Bivalvia)	*A. flos-aquae*, *M. aeruginosa*, *P. agardhii*, *A. circinalis*	MC	ELISA	ND–0.39 [(a)]	
Potamopyrgus antipodarum (Gastropoda)	*A. flos-aquae*, *M. aeruginosa*, *P. agardhii*, *A. circinalis*	MC	ELISA	ND–0.35 [(a)]	
Planorbis planorbis (Gastropoda)	*A. flos-aquae*, *M. aeruginosa*, *P. agardhii*, *A. circinalis*	MC	ELISA	ND–0.15 [(a)]	
Radix auricularia (Gastropoda)	*A. flos-aquae*, *M. aeruginosa*, *P. agardhii*, *A. circinalis*	MC	ELISA	ND–1.04 [(a)]	
Radix ovata (Gastropoda)	*A. flos-aquae*, *M. viridis*, *M. aeruginosa*, *P. agardhii*, *A. circinalis*	MC	ELISA	ND–0.20 [(a)]	
Physella acuta (Gastropoda)	*M. viridis*, *P. agardhii*, *A. flos-aquae*, *M. aeruginosa*, *A. circinalis*	MC	ELISA	ND–242.7 [(a)]	
Aplexa hypnorum (Gastropoda)	No cyanobacteria found	MC	ELISA	ND–0.07 [(a)]	
Bellamya aeruginosa (Gastropoda)	*M. aeruginosa*	MC-LR, -LR-GSH, -LR-Cys	LC-MS	ND–9.70 [(a)]	Zhang *et al.* (2009)[34]
Bellamya aeruginosa (Gastropoda)	*M. aeruginosa*	MC-LR,-RR,-YR	LC-MS	ND–12.2 [(a)]	Zhang *et al.* (2009)[75]
Pomacea patula catemacensis (Gatropoda)	*Cylindrospermopsis catemaco*, *C. philippinensis*	CYN	ELISA, HPLC, LC-MS	ND–0.00335 [(a)]	Berry and Lind (2010)[76]
		STXs		ND–0.00104 [(a)]	
Utterbackia imbecillis (Bivalvia)	Undetermined cyanobacterial blooms	BMAA	HPLC-LC/MS/MS	251–261	Brand *et al.* (2010)[35]
Crassostra virginica (Bivalvia)	Undetermined cyanobacterial blooms	BMAA	HPLC-LC/MS/MS	281–305	
L. stagnalis (Gastropoda)	*P. agardhii*	MC	LC–ESI-MS/MS ELISA	up to 32.4 [(a)] free MC	Lance *et al.* (2010)[77]

Table S1. *Cont.*

L. stagnalis (Gastropoda)	*P. agardhii*	MC	MMPB LC–ESI-MS/MS	up to 37.5 [(a)] bound MC	
Physia acuta (Gastropoda)	Several	MC	ELISA	ND–4.32 [(a)]	Lance *et al.* (2010)[78]
Several gastropod species	Several	MC	ELISA	0.02–1.26 [(a)]	
L. stagnalis (Gastropoda)—Caged experiment	Several	MC	ELISA	0.02–0.32 [(a)]	
Potamopyrgus antipodarum (Gastropoda)—Caged experiment	Several	MC	ELISA	0.01–0.02 [(a)]	

Table S2. Cyanotoxins content in aquatic vertebrates. Concentrations of microcystins are given in equivalents of MC-LR, and concentrations of saxitoxins are given in equivalents of STX. Concentrations represent the range for the different organs/tissues in µg g^{-1} DW. Concentrations in µg g^{-1} WW were converted to µg g^{-1} DW multiplying by 10 or by 5 (fish muscle). MC = microcystins; NOD = nodularin; CYN = cylindrosdpermopsin; STX = saxitoxins; ANTX-a = anatoxin-a; BMAA = β-N-methylamino-L-alanine. HPLC = High Performance Liquid Chromatography; LC-MS = Liquid Chromatography–Mass Spectrometry; ELISA = Enzyme Linked Immunosorbant Assay; PP2A = Phosphatase 2A Inhibition Assay; PP1 = Phosphatase 1 Inhibition Assay; GC-MS = Gas Chromatography–Mass Spectrometry; WW = wet weight; DW = dry weight. ND = not detectable.

Group of organism/Taxa	Trophic guild	Dominant cyanobacteria	Toxin	Analytical method	Concentration µg g^{-1} DW	Reference
**FISH**						
Melanotaenia eachamensis (Melanotaeniidae)	Carnivore	*C. raciborskii*	CYN	HPLC	1.2	Saker and Eaglesham (1999)[22]
Dorosoma cepedianum (Clupeidae)	Omnivore	*M. aeruginosa*	MC	ELISA	2.97–11.91	Babcock-Jackson (2000)[23]
Perca flavescens (Percidae)	Carnivore	*M. aeruginosa*	MC	ELISA	0.46–2.30	
Sander vitreus (Percidae)	Carnivore	*M. aeruginosa*	MC	ELISA	0.50	
Morone spp. (Moronidae)	Carnivore	*M. aeruginosa*	MC	ELISA	0.62–2.33	
Semolitus atromaculatus (Cyprinidae)	Omnivore	*M. aeruginosa*	MC	ELISA	0.60–0.94	
Neogobius melanostomus (Gobiidae)—Field data	Carnivore	*M. aeruginosa*	MC	ELISA	0.48–1.87	
Neogobius melanostomus (Gobiidae)—Lab experiment	Carnivore	*M. aeruginosa*	MC	ELISA	up to 2.82	

Table S2. *Cont.*

Platichthys flesus (Pleuronectidae)	Carnivore	*Nodularia spumigena*	NOD	ELISA/PP1	0.05–0.06	Sipiä *et al.* (2000)[79]
Gadus morhua (Gadidae)	Carnivore	*Nodularia spumigena*	NOD	ELISA/PP1	ND–0.14	
Tilapia rendalli (Cichlidae)	Omnivore	*M. aeruginosa*	MC	HPLC/ELISA	ND–678	Magalhães *et al.* (2001)[80]
Platichthys flesus (Pleuronectidae)	Carnivore	*Aphanizomenon* sp., *N. spumigena*, *Anabaena* sp.	NOD/MC	ELISA	ND–0.40	Sipiä *et al.* (2001)[48]
Platichthys flesus (Pleuronectidae)	Carnivore	*Aphanizomenon flos-aquae*, *N. spumigena*	NOD/MC	ELISA/LC-MS/MS	ND–0.41	Sipiä *et al.* (2002)[49]
Gasterosteus aculeatus (Gasterosteidae)	Planktivore	*N. spumigena*	NOD	ELISA/PP1	0.15–0.80	Engström-Öst *et al.* (2002)[25]
Salmo trutta m. *trutta* L. (Salmonidae)	Carnivore	*N. spumigena*	NOD-like compound	ELISA	0.011–1.60	Kankaanpää *et al.* (2002)[81]
Fish muscle—unidentified species	Unknown	Picoplanktonic cyanobacteria (unidentified)	MC	ELISA	ND–0.02	Magalhães *et al.* (2003)[26]
Oreochromis niloticus (Cichlidae)	Omnivore	*M. aeruginosa*	MC	ELISA	0.4–8.3	Mohamed *et al.* (2003)[82]
Tilapia rendalli (Cichlidae)	Omnivore	*M. aeruginosa*	MC	ELISA	0.02–28.0	Soares *et al.* (2004)[83]
Hypophytalmincthtys molitrix (Cyprinidae)	Phytoplanktivore	*Microcystis viridis*	MC-RR, -LR	HPLC	49.2–115.3	Xie *et al.* (2004)[84]
Jenynsia multidentata (Anablepidae)	Omnivore	Purified toxin	MC-RR	HPLC	0.55–16.0	Cazenave *et al.* (2005)[85]
Corydoras paleatus (Callichthyidae)	Omnivore	Purified toxin	MC-RR	HPLC	0.2–196.3	
Odontesthes bonariensis (Atherinidae)	Carnivore	Undetermined cyanobacterial blooms	MC-RR	HPLC	ND–10.1	
Perca fluviatilis (Percidae)	Carnivore	*M. aeruginosa*, *Planktothrix agadhii*	MC	LC-MS/HPLC/ELISA	17.0–51.0	Ibelings *et al.* (2005)[11]
Gymnocephalus cernua (Percidae)	Omnivore	*M. aeruginosa*, *Planktothrix agadhii*	MC	LC-MS/HPLC/ELISA	9.0–194	
Osmerus eperlanus (Osmeridae)	Zooplanktivore	*M. aeruginosa*, *Planktothrix agadhii*	MC	LC-MS/HPLC/ELISA	59–874	
Platichthys flesus (Pleuronectidae)	Carnivore	*N. spumigena*	TEH/NOD	ELISA/LC-MS	ND–2.230	Kankaanpää *et al.* (2005)[86]
Hypophytalmincthtys molitrix (Cyprinidae)	Phytoplanktivore	*Microcystis* sp., *Anabaena* sp.	MC	HPLC	1.16–137.0	Xie *et al.* (2005)[87]
Parabramis perkinensis (Cyprinidae)	Herbivore	*Microcystis* sp., *Anabaena* sp.	MC	HPLC	0.66–7.5	
Carassius auratus (Cyprinidae)	Omnivore	*Microcystis* sp., *Anabaena* sp.	MC	HPLC	1.8–42.6	

Table S2. *Cont.*

Cyprinus carpio (Cyprinidae)	Omnivore	*Microcystis* sp., *Anabaena* sp.	MC	HPLC	0.65–6.46	
Culter erythropterus (Cyprinidae)	Carnivore	*Microcystis* sp., *Anabaena* sp.	MC	HPLC	2.0–13.7	
Culter ilishaeformis (Cyprinidae)	Carnivore	*Microcystis* sp., *Anabaena* sp.	MC	HPLC	2.73–5.0	
Pseudobagrus fulvidraco (Bagridae)	Carnivore	*Microcystis* sp., *Anabaena* sp.	MC	HPLC	2.0–8.0	
Coilia ectenes (Bagridae)	Carnivore	*Microcystis* sp., *Anabaena* sp.	MC	HPLC	ND–0.91	
Esox lucius (Esocidae)	Carnivore	*N. spumigena*	NOD	Radiolabeled NOD	0.41	Karjalainen *et al.* (2005)[29]
Hypophytalmincthtys molitrix (Cyprinidae)	Phytoplanktivore	*M. aeruginosa*	MC-LR, -RR, -YR	LC-MS	ND–97.48	Chen *et al.* (2006)[88]
Ascipenser gueldenstaedtii (Acipenseridae)—Lake Pamvotis	Carnivore	*M. aeruginosa*	MC	PP2A/ELISA	0.30–1.44	Gkelis *et al.* (2006)[64]
Carassius auratus (Cyprinidae)—Lake Pamvotis	Omnivore	*M. aeruginosa*	MC	PP2A/ELISA	0.12–5.40	
Carassius gibelio (Cyprinidae)—Lake Kastoria	Omnivore	*M. aeruginosa*, *M. flos-aquae*	MC	PP2A/ELISA	0.09–0.72	
Carassius gibelio (Cyprinidae)—Lake Kerkini	Omnivore	*Microcystis* spp.	MC	PP2A/ELISA	0.14–2.80	
Cyprinus carpio (Cyprinidae)—Lake Kerkini	Omnivore	*Microcystis* spp.	MC	PP2A/ELISA	0.09–0.82	
Cyprinus carpio (Cyprinidae)—Lake Pamvotis	Omnivore	*M. aeruginosa*; *A. flos-aquae*	MC	PP2A/ELISA	0.02–1.30	
Cyprinus carpio (Cyprinidae)—Lake Yliki	Omnivore	*Microcystis* spp.	MC	PP2A/ELISA	0.07–0.15	
Perca fluviatilis (Percidae)—Lake Kastoria	Carnivore	*M. aeruginosa*, *M. flos-aquae*	MC	PP2A/ELISA	0.07–1.60	
Rutilus rubilo (Cyprinidae)—Lake Pamvotis	Omnivore	*M. aeruginosa*	MC	PP2A/ELISA	0.02–0.39	
Silurus aristoteles (Siluridae)—Lake Pamvotis	Carnivore	*M. aeruginosa*	MC	PP2A/ELISA	0.09–0.24	
Silurus glanis (Siluridae)—Lake Pamvotis	Carnivore	*M. aeruginosa*	MC	PP2A/ELISA	0.04–0.16	
Oreochromis niloticus (Cichlidae)	Omnivore	*M. aeruginosa*	MC	ELISA/PP2A	0.2–25.0	Mohamed and Hussein (2006)[89]
Rutilus rutilus (Cyprinidae)	Omnivore	*N. spumigena*	NOD	LC-MS/ELISA	ND–0.9	Sipiä *et al.* (2006)[90]
Platichthys flesus (Pleuronectidae)	Carnivore	*N. spumigena*	NOD	LC-MS/ELISA	0.003–1.1	
Lepomis gibbosus (Centrarchidae)—Barbadoes Pond	Zooplanktivore	*Microcystis* sp.	MC	ELISA	0.02–0.06	Smith and Haney (2006)[14]
Lepomis gibbosus (Centrarchidae)—Lab. experiment	Zooplanktivore	*Microcystis* sp.	MC	ELISA	0.01–0.11	

Table S2. *Cont.*

Oncorhyncus myskiss (Salmonidae)—Lake Rotoiti	Carnivore	*Microcystis* sp., *Anabaena* sp.	MC	LC-MS/ELISA	0.04–0.46	Wood *et al.* (2006)[63]
Oncorhyncus myskiss (Salmonidae)—Lake Rotoehu	Carnivore	*Microcystis* sp., *Anabaena* sp.	MC	LC-MS/ELISA	0.28–0.79	
Cyprinus carpio (Cyprinidae)	Omnivore	*M. aeruginosa*, *M. ichtyoblade*, *A. floes-aquae*	MC	ELISA	0.07–1.32	Adamovský *et al.* (2007)[91]
Hypophytalmincthtys molitrix (Cyprinidae)	Phytoplanktivore	*M. aeruginosa*, *M. ichtyoblade*, *A. floes-aquae*	MC	ELISA	0.05–1.24	
Platichthys flesus (Pleuronectidae)	Carnivore	*N. spumigena*	NOD	ELISA	ND–0.47	Mazur-Marzec *et al.* (2007)[69]
Cyprinus carpio (Cyprinidae)	Omnivore	*Anabaena* sp. (ANA 37)	ANTX-a	HPLC	0.03–0.77 (a)	Osswald *et al.* (2007)[92]
Gasterosteus aculeatus (Gasterosteidae)	Planktivore	*N. spumigena*	NOD	LC-MS	0.003–0.7	Sipiä *et al.* (2007)[93]
Clupea harengus(Clupeidae)	Planktivore	*N. spumigena*	NOD	LC-MS	ND–0.09	
Carassius auratus auratus (Cyprinidae)	Omnivore	*Microcystis* spp.	MC	ELISA/LC-MS	ND–3.64	Song *et al.* (2007)[33]
Carassius auratus (Cyprinidae)	Omnivore	*Microcystis* spp., *A. flos-aquae*	MC-RR	HPLC	0.49–0.82	Xie *et al.* (2007)[70]
Cyprinus carpio (Cyprinidae)	Omnivore	*Microcystis* spp., *A. flos-aquae*	MC-RR	HPLC	0.27–2.06	
Silurus glanis (Siluridae)	Omnivore	*Microcystis* spp., *A. flos-aquae*	MC-RR	HPLC	0.14	
Hypophytalmincthtys molitrix (Cyprinidae)	Phytoplanktivore	*M. aeruginosa*	MC-RR, -LR	HPLC-MS	13.5–91.0	Zhang *et al.* (2007)[94]
Clupea harengus membras (Clupeidae)	Planktivore	*N. spumigena*	NOD	ELISA	up to 0.22	Karjalainen *et al.* (2008)[17]
Sprattus sprattus (Clupeidae)	Planktivore	*N. spumigena*	NOD	ELISA	up to 0.10	
Gasterosteus aculeatus (Gasterosteidae)	Planktivore	*N. spumigena*	NOD	ELISA	up to 0.80	
Perca flavescens (Percidae)	Carnivore	*M. aeruginosa*	MC	ELISA	0.017–1.18	Wilson *et al.* (2008)[95]
Tilapia rendalli (Cichlidae) Furnas Reservoir	Omnivore	*Pseudanabaena mucicola*, *Microcystis panniformis*, *M. aeruginosa*	MC	PP1-HPLC	0.005–24	Deblois *et al.* (2008)[96]
Oreochromis niloticus (Cichlidae)—Furnas Reservoir	Omnivore		MC	PP1-HPLC	0.06–112	
Oreochromis niloticus (Cichlidae)—Funil Reservoir	Omnivore	*M. panniformis*, *M. viridis*, *P. mucicola*, *Anabaena* cf. *crassa*	MC	PP1-HPLC	0.02–321	

Table S2. *Cont.*

Hypophytalmincthtys molitrix (Cyprinidae)	Phytoplanktivore	*Microcystis* spp.	MC-LR, -RR, -YR	LC-MS	ND–5.36	Chen *et al.* (2009)[97]
Carassius auratus (Cyprinidae)	Omnivore	*Microcystis* spp.	MC-LR, -RR, -YR	LC-MS	0.04–0.27	
Culter ilishaeformis (Cyprinidae)	Carnivore	*Microcystis* spp.	MC-LR, -RR, -YR	LC-MS	ND–0.01	
Cyprinus carpio (Cyprinidae)	Omnivore	*Microcystis* spp.	MC-LR, -RR, -YR	LC-MS	0.002–0.064	
Platichthys flesus (Pleuronectidae)	Carnivore	*N. spumigena*	NOD	HPLC	ND–0.78	Persson *et al.* (2009)[98]
Platichthys flesus (Pleuronectidae)	Carnivore	*N. spumigena*	NOD	LC-MS	0.045–0.220	Vuorinen *et al.* (2009)[99]
Hypophytalmincthtys molitrix (Cyprinidae)	Phytoplanktivore	*M. aeruginosa*	MC-LR,-LR-GSH, -LR-Cys	LC-MS	ND–29.1	Zhang *et al.* (2009)[34]
Coilia ectenes (Bagridae)	Carnivore	*M. aeruginosa*	MC-LR, -RR, -YR	LC-MS	ND–0.0447	Zhang *et al.* (2009)[100]
Culter ilishaeformis (Cyprinidae)	Carnivore	*M. aeruginosa*	MC-LR, -RR, -YR	LC-MS	ND–0.0581	
Neosalanx taihuensis (Salangidae)	Carnivore	*M. aeruginosa*	MC-LR, -RR, -YR	LC-MS	ND–0.00756	
Hypophytalmincthtys molitrix (Cyprinidae)	Phytoplanktivore	*M. aeruginosa*	MC-LR, -RR, -YR	LC-MS	ND–4.19	
Carassius auratus (Cyprinidae)	Omnivore	*M. aeruginosa*	MC-LR, -RR, -YR	LC-MS	ND–0.2064	
Carassius gibelio (Cyprinidae)—Lakes in Greece	Omnivore	Several species	MC	ELISA	0.011–35.6	Papadimitriou *et al.* (2010)[101]
Odontesthes bonariensis (Atherinidae)	Carnivore	*Planktothrix agardhii*, *Anabaena* sp., *Microcystis* sp.	MC-LR, -RR, -YR, -LA	HPLC-MS/MS	ND–0.673	Amé *et al.* (2010)[102]
Lutjanus griseus (Lutjanidae)	Carnivore	Cyanobacterial blooms	BMAA	HPLC-C/MS/MS	ND–188	Brand *et al.* (2010)[35]
Mojarra (Gerreidae)	Carnivore	Cyanobacterial blooms	BMAA		ND–567	
Sphoeroides spengleri (Tetraodontidae)	Carnivore	Cyanobacterial blooms	BMAA		ND–455	
Sphoeroides parvus (Tetraodontidae)	Carnivore	Cyanobacterial blooms	BMAA		194–7351	
Acanthostracion quadricornis (Ostraciidae)	Omnivore	Cyanobacterial blooms	BMAA		34–47	
Archosargus rhomboidalis (Sparidae)	Omnivore	Cyanobacterial blooms	BMAA		484–2349	
Haemulon parra (Haemulidae)	Carnivore	Cyanobacterial blooms	BMAA		203–722	
Haemulon sciurus (Haemulidae)	Carnivore	Cyanobacterial blooms	BMAA		1723–3776	
Amia calva (Amiidae)	Carnivore	Cyanobacterial blooms	BMAA		554–2559	
Atractosteus spatula (Lepisosteidae)	Carnivore	Cyanobacterial blooms	BMAA		797–2290	
Micropterus salmoides (Centrarchidae)	Carnivore	Cyanobacterial blooms	BMAA		949–2388	

Table S2. *Cont.*

Geophagus brasiliensis (Cichlidae)	Omnivore	*C. raciborskii*	STXs	HPLC	0.12–0.20	Clemente *et al.* (2010)[103]
Cyprinus carpio larvae (Cyprinidae)	Omnivore	*M. aeruginosa*	MC-LR, -RR, -YR, -FR,-WR	HPLC-MS	0.17–0.27	El Ghazali *et al.* (2010)[104]
Cyprinus carpio larvae (Cyprinidae)	Omnivore	*M. aeruginosa*	MC-LR, -RR, -YR, -FR,-WR	HPLC-MS	0.22–0.55	El Ghazali *et al.* (2010)[105]
Lates niloticus (Latidae)	Carnivore	No information	MC-LR, -RR, -YR	HPLC-MS/MS	0.0097–0.28	Nyakairu *et al.* (2010)[106]
Oreochromis niloticus (Cichlidae)—Lake Mburo	Omnivore	No information	MC-LR, -RR, -YR	HPLC-MS/MS	0.731–13.12	
Oreochromis niloticus (Cichlidae)—Murchison Bay	Omnivore	No information	MC-LR, -RR, -YR	HPLC-MS/MS	0.048–14.79	
Morone saxatilis (Moronidae)	Zooplanktivore	*Microcystis* sp.	MC	PP2A	1.04–2.92	Lehman *et al.* (2010)[21]
Menidia audens (Atherinidae)	Zooplanktivore	*Microcystis* sp.	MC	PP2A	0.30–1.98	
Chirostoma spp. (Atherinidae)	Zooplanktivore	*Aphanizomenon*; *Microcystis*	MC	ELISA	0.185	Berry *et al.* (2011)[107]
Cyprinus carpio (Cyprinidae)	Omnivore	*Aphanizomenon*; *Microcystis*	MC	ELISA	0.05–0.94	
Goodea sp. (Goodeidae)	Phytoplanktivore	*Aphanizomenon*; *Microcystis*	MC	ELISA	1.57–8.67	
**AMPHIBIANS**						
Rana eperotica (Ranidae)—Lake Pamvotis	Carnivore	*M. aeruginosa*	MC	PP2A/ELISA	0.26–0.47	Gkelis *et al.* (2006)[64]
Rana ridibunda (Ranidae)—Lake Gallikos	Carnivore	*M. aeruginosa*	MC	PP2A/ELISA	0.37–0.47	
Bufo marinus (Bufonidae) tadpoles	Phytoplanktivore	*Cylindrospermopsis raciborskii*	CYN	HPLC/MS-MS	0.61–8.95	White *et al.* (2007)[108]
**REPITILES**						
Emys orbicularis (Emydidae)	Carnivore	*Microcystis* spp.	MC-LR, -RR, -YR	PP2A/ Limieux-GC-MS	0.001–37.2	Nasri *et al.* (2008)[109]
Mauremys leprosa (Emydidae)	Omnivore	*Microcystis* spp.	MC-LR, -RR, -YR	PP2A/ Limieux-GC-MS	0.02–1,193	
Pelodiscus sinensis (Tryonichidae)	Carnivore	*Microcystis* spp.	MC-LR, -RR, -YR	LC-MS	0.011–0.021	Chen *et al.* (2009)[97]

Table S2. *Cont.*

**BIRDS**						
Phoeniconaias minor (Phoenocopteridae)	Omnivore	*Synechococcus bigranulatus*, *Spirulina subsalsa*, *Phormidium terebriformis*, *Oscillatoria willei*	MC<br>ANTX-a	HPLC/MS	0.36<br>7.62	Krienitz *et al.* (2003)[110]
Somateria mollissima (Anatidae)	Carnivore	*N. spumigena*	NOD	LC-MS/ELISA	0.003–0.180	Sipiä *et al.* (2003)[111]
Somateria mollissima (Anatidae)	Carnivore	*N. spumigena*	NOD	LC-MS/ELISA	ND–0.198	Sipiä *et al.* (2006)[90]
Somateria mollissima (Anatidae)	Carnivore	*N. spumigena*	NOD	LC-MS/ELISA	0.003–0.052	Sipiä *et al.* (2008)[71]
Coturnix coturnix japonica (Phasianidae)	Omnivore	*Microcystis* sp.	MC	ELISA	0.0043–0.437	Pašková *et al.* (2008)[112]
Anas platyrhynchos (Anatidae)	Omnivore	*Microcystis* spp.	MC-LR, -RR,-YR	LC-MS	0.009–0.051	Chen *et al.* (2009)[97]
Nycticorax nycticorax (Ardeidae)	Omnivore	*Microcystis* spp.	MC-LR, -RR,-YR	LC-MS	0.003–0.082	

References

1. Watanabe, M.M.; Kaya, K.; Takamura, N.J. Fate of the toxic cyclic hepatopeptides, the microcystins, from blooms of *Microcystis* (Cyanobacteria) in a hypereutrophic lake. *Phycology* **1992**, *28*, 761–767.
2. Kotak, B.G., Zurawell, R.W.; Prepas, E.E.; Holmes, C.F.B. Microcystin-LR concentrations in aquatic food web compartments from lakes of varying trophic status. *Can. J. Fish. Aquat. Sci.* **1996**, *53*, 1974–1985.
3. Thorstrup, L.; Christoffersen, K. Accumulation of microcystin in *Daphnia magna* feeding on toxic *Microcystis*. *Arch. Hydrobiol*. **1999**, *145*, 447–467.
4. Mohamed, Z.A. Accumulation of cyanobacterial hepatotoxins by *Daphnia* in some Egyptian irrigation canals. *Ecotoxicol. Environ. Saf. 2001*, *50*, 4–8.
5. Ferrão-Filho, A.S.; Kozlowsky-Suzuki, B.; Azevedo, S.M.F.O. Accumulation of microcystins by a tropical zooplankton community. *Aquat. Toxicol*. **2002**, *59*, 201–208.
6. Lehtiniemi, M.; Engström-Ost, J.; Karjalainen, M.; Kozlowsky-Suzuki, B.; Vitasalo, M. Fate of cyanobacterial toxins in the pelagic food web: transfer to copepods or to faecal pellets? *Mar. Ecol. Progr. Ser.* **2002**, *241*, 13–21.
7. Kozlowsky-Suzuki, B.; Karjalainen, M.; Lehtiniemi, M.; Engström-Ost, J.; Koski, M.; Carlsson, P. Feeding, reproduction and toxin accumulation by the copepods *Acartia bifilosa* and *Eurytemora affinis* in the presence of the toxic cyanobacterium *Nodularia spumigena*. *Mar. Ecol. Progr. Ser.* **2003**, *249*, 237–249.
8. Karjalainen, M.; Reinikainen, M.; Lindvall, F.; Spoof, L.; Meriluoto, J.A.O. Uptake and accumulation of dissolved radiolabeled nodularin in Baltic Sea zooplankton. *Environ. Toxicol.* **2003**, *18*, 52–60.
9. Nogueira, I.C.G.; Saker, M.L.; Pflugmacher, S.; Wiegand, C.; Vasconcelos, V.M. Toxicity of the cyanobacterium *Cylindrospermopsis raciborskii* to *Daphnia magna*. *Environ. Toxicol*. **2004**, *19*, 453–459.
10. Nogueira, I.C.G.; Pereira, P.; Dias, E.; Pflugmacher, S.; Wiegand, C.; Franca, S.; Vasconcelos, V.M. Accumulation of paralytic shellfish toxins (PST) from the cyanobacterium *Aphanizomenon issatschenkoi* by cladoceran *Daphnia magna*. *Toxicon* **2004**, *44*, 773–780.
11. Ibelings, B.W.; Bruning, K.; Jonge, J.; Wolfstein, K.; Dionisio, L.M.; Postma J.; Burger, T. Distribuition of microcystins in a lake foodweb: no evidence for biomagnification. *Microb. Ecol*. **2005**, *49*, 487–500.
12. Lehman, P.W.; Boyer, G.; Hall, C.; Waller, S.; Gehrts, K.D. Distribution and toxicity of a new colonial *Microcystis aeruginosa* bloom in the San Francisco Bay Estuary, California. *Hydrobiologia* **2005**, *541*, 87–99.
13. Karjalainen, M.; Kozlowsky-Suzuki, B.; Lehtiniemi, M.; Engström-Öst, J.; Kankaanpää, H.; Viitasalo, M. Nodularin accumulation during cyanobacterial blooms and experimental depuration in zooplankton. *Mar. Biol*. **2006**, *148*, 683–691.
14. Smith, J.L.; Haney, J.F. Foodweb transfer, accumulation and depuration of microcystins, a cyanobacterial toxin, in pumpkinmsees sunfish (*Lepomis gibbosus*). *Toxicon* **2006**, *48*, 580–589.

15. Oberhaus, L.; Gélinas, M.; Pinel-Alloul, B.; Ghadouani, A.; Humbert, J.-F. Grazing of two toxic *Planktothrix* species by *Daphnia pulicaria*: potential for bloom control and transfer of microcystins. *J. Plank. Res.* **2007**, *29*, 827–838.
16. Rocha, M.I.A. Estudo da Variação Sazonal do Fitoplâncton e Zooplâncton e da Concentração de Microcistinas nesses dois Níveis Tróficos no Reservatório do Funil (Resende-RJ). M.Sc. Thesis, Instituto de Biofísica Carlos Chagas Filho, Universidade Federal do Rio de Janeiro, Rio de Janeiro, Brazil, 2005; pp. 1–60.
17. Karjalainen, M.; Pääkkönen, J.-P.; Peltonen, H.; Sipiä, V.; Valtonen, T.; Viitasalo, M. Nodularin concentrations in Baltic Sea zooplankton and fish during a cyanobacterial bloom. *Mar. Biol.* **2008**, *155*, 483–491.
18. Lehman, P.W.; Boyer, G.; Satchwell, M.; Waller, S. The influence of environmental conditions on the seasonal variation of *Microcystis* cell density and microcystins concentration in San Francisco Estuary. *Hydrobiologia* **2008**, *600*, 187–204.
19. Gorokhova, E.; Engström-Öst, J. Toxin concentration in *Nodularia spumigena* is modulated by mesozooplankton grazers. *J. Plankton Res.* **2009**, *31*, 1235–1247.
20. Sopanen, S.; Uronen, P.; Kuuppo, P.; Svensen, C.; Rühl, A.; Tamminen, T.; Granéli, E.; Legrand, C. Transfer of nodularin to the copepod *Eurytemora affinis* through the microbial food web. *Aquat. Microb. Ecol.* **2009**, *55*, 115–130.
21. Lehman, P.W.; Teh, S.J.; Boyer, G.; Nobriga, M.L.; Bass, E.; Hogle, C. Initial impacts of *Microcystis aeruginosa* blooms on the aquatic food web in the San Francisco Estuary. *Hydrobiologia* **2010**, *637*, 229–248.
22. Saker, M.L.; Eaglesham, G.K. The accumulation of cylindrospermopsin from the cyanobacterium *Cylindrospermopsis raciborskii* in tissues of the Redclaw crayfish *Cherax quadricarinatus*. *Toxicon* **1999**, *37*, 1065–1077.
23. Babcock-Jackson, L. Toxic Microcystis in western Lake Erie: Ecotoxicological relationships with three non-indigenous species increase risks to the aquatic community. Ph.D. Thesis, Ohio State University, Columbus, Ohio, USA, 2000; pp. 1–189.
24. Vasconcelos, V.; Oliveira, S.; Teles, F.O. Impact of a toxic and a non-toxic strain of *Microcystis aeruginosa* on the crayfish *Procambarus clarkii*. *Toxicon* **2001**, *39*, 1461–1470.
25. Engström-öst, J.; Lehtinienmi, M.; Green, S.; Kozlowsky-Suzuki, B., Viitassalo, M. Does cyanobacterial toxin accumulate in mysid shrimps and fish via copepods? *J. Exp. Biol. Ecol.* **2002**, *276*, 95–107.
26. Magalhães, V.F.; Marinho, M.M.; Domingos, P.; Oliveira, A.C.; Costa, S.M.; Azevedo, L.O.; Azevedo, S.M.F.O. Microcystins (cyanobacteria hepatotoxins) bioaccumulation in fish and crustaceans from Sepetiba Bay (Brazil, RJ). *Toxicon* **2003**, *42*, 289–295.
27. Chen, J.; Xie, P. Tissue distributions and seasonal dynamics of the hepatotoxic microcystins-LR and -RR in two freshwater shrimps, *Palaemon modestus* and *Macrobrachium nipponensis*, from a large shallow, eutrophic lake of the subtropical China. *Toxicon* **2005**, *45*, 615–625.
28. Kankaanpää, H.T.; Holliday, J.; Schröder, H.; Goddard, T.J.; von Fister, R.; Carmichael, W.W. Cyanobacteria and prawn farming in northern New South Wales, Australia—a case study on cyanobacteria diversity and hepatotoxin bioaccumulation. *Toxicol. Appl. Pharmacol.* **2005**, *203*, 243–256.

29. Karjalainen, M.; Reinikainen, M.; Spoof, L.; Meriluoto, J.A.O.; Sivonen, K.; Vitasalo, M. Trophic transfer of cyanobacterial toxins from zooplankton to planktivores: consequences for pike larvae and mysid shrimps. *Environ. Toxicol.* **2005**, *20*, 354–362.
30. Korpinen, S.; Karjalainen, M.; Viitasalo, M. Effects of cyanobacteria on survival and reproduction of the littoral crustacean *Gammarus zaddachi* (Amphipoda). *Hydrobiologia* **2006**, *559*, 285–295.
31. Dewes, L.J.; Sandrini, J.Z.; Monserrat, J.M.; Yunes, J.M. Biochemical and physiological responses after exposure to microcystins in the crab *Chasmagnathus granulatus* (Decapoda, Brachyura). *Ecotoxicol. Environ. Saf.* **2006**, *65*, 201–208.
32. Dewes, L.J.; Monserrat, J.M.; Iharai, P.M.; Yunes, J.M. Toxin accumulation and biochemical alterations in the estuarine crab *Neohelice granulata* (Decapoda, Brachyura) after exposure to microcystins. *J. Braz. Soc. Ecotoxicol.* **2007**, *2*, 203–209.
33. Song, L.; Chen, W.; Peng, L.; Wan, N.; Gan, N.; Zhang, X. Distribution and bioaccumulation of microcystins in water columns: a systematic investigation into the environmental fate and the risks associated with microcystins in Meiliang Bay, Lake Taihu. *Water Res.* **2007**, *41*, 2853–2864.
34. Zhang, D.; Xie, P.; Chen, J.; Dai, M.; Qiu, T.; Liu, Y.; Liang, G. Determination of microcystin-LR and its metabolites in snail (*Bellamya aeruginosa*), shrimp (*Macrobrachium nipponensis*) and silver carp (*Hypophthalmichthys molitrix*) from Lake Taihu, China. *Chemosphere* **2009**, *76*, 974–981.
35. Brand, L.E.; Pablo, J.; Compton, A.; Hammerschlag, N.; Mash, D.C. Cyanobacterial blooms and the occurrence of the neurotoxin, beta-*N*-methylamino-L-alanine (BMAA), in South Florida aquatic food webs. *Harmful Algae* **2010**, *9*, 620–635.
36. Garcia, A.C.; Bargu, S.; Dash, P.; Rabalais, N.N.; Sutor, M.; Morrison, W.; Walker, N.D. Evaluating the potential risk of microcystins to blue crab (*Callinectes sapidus*) fisheries and human health in a eutrophic estuary. *Harmful Algae* **2010**, *9*, 134–143.
37. Sasner, J.J.; Ikawa, M.; Foxall, T.L. Studies on aphanizomenon and microcystis toxins. In *Seafood Toxins*; Ragelis, E.P., Ed.; ACS Publications: Washington, DC, USA, 1984; pp. 391–406.
38. Eriksson, J.E.; Meriluoto, J.A.O.; Lindholm, T. Accumulation of a peptide toxin from the cyanobacterium *Oscillatoria agardhii* in the freshwater mussel *Anadonta cygnea*. *Hydrobiologia* **1989**, *183*, 211–216.
39. Lindholm, T.; Eriksson, J.E.; Meriluoto, J.A.O. Toxic cyanobacteria and water quality problems—examples from a eutrophic lake on Åland. South West Finland. *Water Res.* **1989**, *23*, 481–486.
40. Chen, D.Z.X.; Boland, M.P.; Smillie, M.A.; Klix, H.; Ptak, C.; Andersen, R.J.; Holmes, C.F.B. Identification of protein phosphatase inhibitors of the microcystins class in the marine environment. *Toxicon* **1993**, *31*, 1407–1414.
41. Negri, A.P.; Jones, G.J. Bioaccumulation of paralytic shellfish poisoning (PSP) toxins from the cyanobacterium *Anabaena circinalis* by the freshwater mussels *Alathrya condola*. *Toxicon* **1995**, *33*, 667–678.
42. Vasconcelos, V. Uptake and depuration of the heptapeptide toxin microcystin-LR in *Mytilus galloprovincialis*. *Aquat. Toxicol.* **1995**, *32*, 227–237.

43. Prepas, E.E.; Kotak, B.G.; Campbell, L.M.; Evans, J.C.; Hrudey, S.E.; Holmes, C.F.B. Accumulation and elimination of cyanobacterial hepatotoxins by the freshwater clam *Anodonta grandis simpsoniana*. *Can. J. Fish. Aquat. Sci.* **1997**, *54*, 41–46.
44. Watanabe, M.F.; Park, H.-D.; Kondo, F.; Harada, K.-I.; Hayashi, H.; Okino, T. Identification and estimation of microcystins in freshwater mussels. *Nat. Toxins* **1997**, *5*, 31–35.
45. Williams, D.E.; Dawe, S.C.; Kent, M.L.; Andersen, R.J.; Craig, M.; Holmes, C.F.B. Bioaccumulation and clearance of microcystins from salt water mussels, *Mytilus edulis*, and *in vivo* evidence for covalently bound microcystins in mussel tissues. *Toxicon* **1997**, *35*, 1617–1625.
46. Amorim, A.; Vasconcelos, V. Dynamics of microcystins in the mussel *Mylilus galloprovincialis*. *Toxicon* **1999**, *37*, 1041–1052.
47. Zurawell, R.W.; Kotak, B.G.; Prepas, E.E. Influence of lake trophic status on the occurrence of microcystin-LR in the tissue of pulmonate snails. *Freshw. Biol.* **1999**, *42*, 707–718.
48. Sipiä, V.; Kankaanpää, H.T.; Flinkman, J.; Lahti, K.; Meriluoto, J.A.O. Time-dependent accumulation of cyanobacterial hepatotoxins in flounders (*Platichthys flesus*) and mussels (*Mytilus edulis*) from the Northern Baltic Sea. *Environ. Toxicol.* **2001**, *16*, 330–336.
49. Sipiä, V.; Kankaanpää, H.T.; Pflugmacher, S.; Flinkman, J.; Furey, A.; James, K.J. Bioaccumulation and detoxification of nodularin in tissues of flounder (*Platichthus flesus*), mussels (*Mytilus edulis*, *Dreissena polymorpha*), and clams (*Macoma balthica*) from the northern Baltic Sea. *Ecotoxicol. Environ. Saf.* **2002**, *53*, 305–311.
50. Yokoyama, A.; Park, H.-D. Mechanism and prediction for contamination of freshwater bivalve (Unionidae) with the cyanobacterial toxin, microcystin, in the hypereutrophic Lake Suwa, Jpn. *Environ. Toxicol.* **2002**, *17*, 424–433.
51. Lehtonen, K.K.; Kankaanpää, H.; Leiniö, S.; Sipiä, V.O.; Pflugmacher, S.; Sandberg-Kilpi, E. Accumulation of nodularin-like compounds from the cyanobacterium *Nodularia spumigena* and changes in acetylcholinesterase activity in the clam *Macoma balthica* during short-term laboratory exposure. *Aquat. Toxicol.* **2003**, *64*, 461–476.
52. Ozawa, K.; Yokoyama, A.; Ishikawa K.; Kumagai K.; Watanabe, M.F.; Park, H.D. Accumulation and depuration of microcystin produced by the cyanobacterium *Microcystis* in a freshwater snail. *Limnology* **2003**, *4*, 131–138.
53. Yokoyama, A.; Park, H.-D. Depuration kinetics and persistence of the cyanobacterial toxin, microcystins-LR, in the freshwater bivalve *Unio douglasiae*. *Environ. Toxicol.* **2003**, *18*, 61–67.
54. Dionisio-Pires, L.M.D.; Karlsson, K.M.; Meriluoto, J.A.O.; Kardinaal, E.; Visser, P.M.; Siewertsen, K.; VanDonk, E.; Ibelings, B.W. Assimilation and depuration of microcystin-LRby the zebra mussel, *Dreissena polymorpha*. *Aquat. Toxicol.* **2004**, *69*, 385–396.
55. Negri, A.P.; Bunter, O.; Jones, B.; Llewellyn, L. Effects of the bloom-forming alga *Trichodesmium erythraeum* on the pearl oyster *Pinctada maxima*. *Aquaculture* **2004**, *232*, 91–102.
56. Pereira, P.; Dias, E.; Franca, S.; Pereira, E.; Carolino, M.; Vasconcelos, V. Accumulation and depuration of cyanobacterial paralytic shellfish toxins by the freshwater mussel *Anodonta cygnea*. *Aquat. Toxicol.* **2004**, *68*, 339–350.
57. Saker, M.L.; Metcalf, J.S.; Codd, G.A.; Vasconcelos, V.M. Accumulation and depuration of the cyanobacterial toxin cylindrospermopsin in the freshwater mussel *Anodonta cygnea*. *Toxicon* **2004**, *43*, 185–194.

58. Chen, J.; Xie, P. Seasonal dynamics of the hepatotoxic Microcystins in various organs of four freshwater bivalves from the large eutrophic Lake Taihu of subtropical China and the risk to human consumption. *Environ. Toxicol.* **2005**, *20*, 572–584.
59. Chen, J.; Xie, P.; Guo, L.; Zheng, L.; NI, L. Tissue distributions and seasonal dynamics of the hepatotoxic microcystins-LR and –RR in in a freshwater snail (*Bellamya aeruginosa*) from a large shallow, eutrophic lake of the subtropical China. *Environ. Pollut.* **2005**, *134*, 423–430.
60. Gérard, C.; Brient, L.; Rouzic, B.L. Variation in the response of juvenile and adult gastropods (*Lymnaea stagnalis*) to cyanobacterial toxin (microcystin-LR). *Environ. Toxicol.* **2005**, *20*, 592–596.
61. Svensen, C.; Strogyloudi, E.; Wexels Riser, C.; Dahlmann, J.; Legrand, C.; Wassmann, P.; Granéli, E., Pagou, K. Reduction of cyanobacterial toxins through coprophagy in *Mytilus edulis*. *Harmful Algae* **2005**, *4*, 329–336.
62. Strogyloudi, E.; Giannakourou, A.; Legrand, C.; Ruehl, A., Granéli, E. Estimating the accumulation and transfer of *Nodularia spumigena* toxins by the blue mussel *Mytilus edulis*: An appraisal from culture and mesocosm experiments. *Toxicon* **2006**, *48*, 359–372.
63. Wood, S.A.; Briggs, L.R.; Sprosen, J.; Ruck, J.G.; Wear, R.G.; Holland, P.T.; Bloxham, M. Changes in concentrations of microcystins in rainbow trout, freshwater mussels, and cyanobacteria in Lakes Rotoiti and Rotoehu. *Environ. Toxicol.* **2006**, *21*, 205–222.
64. White, S.H.; Duivenvoorden, L.J.; Fabbro, L.D.; Eaglesham, G.K. Influence of intracellular toxin concentrations on cylindrospermopsin bioaccumulation in a freshwater gastropod (*Melanoides tuberculata*). *Toxicon* **2006**, *47*, 497–509.
65. Gkelis, S.; Lanaras, T.; Sivonen, K. The presence of microcystins and other cyanobacterial bioactive peptides in aquatic fauna collected from Greek freshwaters. *Aquat. Toxicol.* **2006**, *78*, 32–41.
66. Lance, E.; Brient, L.; Bormans, M.; Gérard, C. Interactions between cyanobacteria and gastropods I. Ingestion of toxic *Planktothrix agardhii* by *Lymnaea stagnalis* and the kinetics of microcystin bioaccumulation and detoxification. *Aquat. Toxicol.* **2006**, *79*, 140–148.
67. Chen, J.; Xie, P. Microcystin accumulation in freshwater bivalves from Lake Taihu, China, and the risk to human consumption. *Environ. Toxicol. Chem.* **2007**, *26*, 1066–1073.
68. Kankaanpää, H.; Leiniö, S.; Olin, M.; Sjövall, O.; Meriluoto, J., Lehtonen, K.K. Accumulation and depuration of cyanobacterial toxin nodularin and biomarker responses in the mussel *Mytilus edulis*. *Chemosphere* **2007**, *68*, 1210–1220.105.
69. Mazur-Marzec, H.; Tyminska, A.; Szafranek, J.; Plinski, M. Accumulation of nodularin in sediments, mussels, and fish from the Gulf of Gdansk, southern Baltic Sea. *Environ. Toxicol.* **2007**, *22*, 101–111.
70. Xie, L.; Yokoyama, A.; Nakamura, K.; Park, H. Accumulation of microcystins in various organs of the freshwater snail *Sinotaia histrica* and three fishes in a temperate lake, the eutrophic Lake Suwa, Japan. *Toxicon* **2007**, *49*, 646–652.
71. Lance, E.; Bugajny, E.; Bormans, M.; Gérard, C. Consumption of toxic cyanobacteria by *Potamopyrgus antipodarum* (Gastropoda, Prosobranchia) and consequences on life traits and microcystin accumulation. *Harmful Algae* **2008**, *7*, 464–472.

72. Sipiä, V.; Neffling, M.R.; Metcalf, J.; Nybom, S.; Meriluoto, J.; Codd, G. Nodularin in feathers and liver of eiders (*Somateria mollissima*) caught from the western Gulf of Finland in June–September 2005. *Harmful Algae* **2008**, *7*, 99–105.
73. Fernandes, S.; Welker, M.; Vasconcelos, V.M. Changes in the GST activity of the mussel *Mytilus galloprovincialis* during exposure and depuration of microcystins. *J. Exp. Zool.* **2009**, *311A*, 226–230.
74. Gérard, C.; Poullain, V.; Lance, E.; Acou, A.; Brient, L.; Carpentier, A. Influence of toxic cyanobacteria on community structure and microcystin accumulation of freshwater molluscs. *Environ. Pollut.* **2009**, *157*, 609–617.
75. Zhang, D. ; Xie, P.; Liu, Y.; Chen, J.; Wen, Z. Spatial and temporal variations of microcystins in hepatopancreas of a freshwater snail from Lake Taihu. *Ecotoxicol. Environ. Saf.* **2009**, *72*, 466–472.
76. Berry, J.P.; Lind, O. First evidence of "paralytic shellfish toxins" and cylindrospermopsin in a Mexican freshwater system, Lago Catemaco, and apparent bioaccumulation of the toxins in "tegogolo" snails (*Pomacea patula catemacensis*). *Toxicon* **2010**, *55*, 930–938.
77. Lance, E.; Neffling, M.R.; Gérard, C.; Meriluoto, J.; Bormans, M. Accumulation of free and covalently bound microcystins in tissues of *Lymnaea stagnalis* (Gastropoda) following toxic cyanobacteria or dissolved microcystin-LR exposure. *Environ. Pollut.* **2010**, *158*, 674–680.
78. Lance, E.; Brient, L.; Carpentier, A.; Acou, A.; Marion, L.; Bormans, M.; Gérard, C. Impact of toxic cyanobacteria on gastropods and microcystin accumulation in a eutrophic lake (Grand-Lieu, France) with special reference to *Physa* (=*Physella*) *acuta*. *Sci. Total Environ.* **2010**, *408*, 3560–3568.
79. Sipiä, V.; Kankaanpää, H.; Lahti, K.; Carmichael, W.W.; Meriluoto, J. Detection of nodularin in flounders and cod from the Baltic Sea. *Environ. Toxicol.* **2000**, *16*, 121–126.
80. Magalhães, V.F.; Soares, R.M.; Azevedo, S.M.F.O. Microcystin contamination in fish from the Jacarepaguá Lagoon (Rio de Janeiro, Brazil): ecological implication and humam health risk. *Toxicon* **2001**, *39*, 1077–1085.
81. Kankaanpää, H.; Vuorinen, P.J.; Sipia, V.; Keinanen, M. Acute effects and bioaccumulation of nodularin in sea trout (*Salmo trutta* L.) exposed orally to *Nodularia spumigena* under laboratory conditions. *Aquat. Toxicol.* **2002**, *61*, 155–168.
82. Mohamed, Z.A.; Carmichael, W.W.; Hussein, A.A. Estimation of microcystins in the freshwater fish *Oreochromis niloticus* in an Egyptian fish farm containing a *Microcystis* bloom. *Environ. Toxicol.* **2003**, *18*, 137–141.
83. Soares, R.M.; Magalhães, V.F.; Azevedo, S.M.F.O. Accumulation and depuration of microcystins (cyanobacteria hepatotoxins) in *Tilapia rendalli* (Cichlidae) under laboratory conditions. *Aquat. Toxicol.* **2004**, *70*, 1–10.
84. Xie, L.; Xie, P.; Ozawa, K.; Honma, T.; Yokoyama, A.; Park, H.-D. Dynamics of microcystins-LR and -RR in the phytoplanktivorous silver carp in a sub-chronic toxicity experiment. *Environ. Pollut.* **2004**, *127*, 431–439.

85. Cazenave, J.; Wunderlin, D.A.; Bistoni, M.A.; Amé, M.V.; Krause, E.; Pflugmacher, S.; Wiegand, C. Uptake, tissue distribution and accumulation of microcystin-RR in *Corydoras paleatus*, *Jenynsia multidentata* and *Odontesthes bonariensis* A field and laboratory study. *Aquat. Toxicol.* **2005**, *75*, 178–190.
86. Kankaanpää, H.; Turunen, A.K.; Karlsson, K.; Bylund, G.; Meriluoto, J.; Sipiä, V. Heterogeneity of nodularin bioaccumulation in northern Baltic Sea flounders in 2002. *Chemosphere* **2005**, *59*, 1091–1100.
87. Xie, L.; Xie, P.; Guo, L.; Li, L.; Miyabara, Y.; Park, H. Organ distribution and bioaccumulation of microcystins in freshwater fish at different trophic levels from the eutrophic Lake Chaohu, China. *Environ. Toxicol.* **2005**, *20*, 293–300.
88. Chen, J.; Xie, P.; Zhang, D.W.; Ke, Z.X.; Yang, H. *In situ* studies on the bioaccumulation of microcystins in the phytoplanktivorous silver carp (*Hypophthalmichthys molitrix*) stocked in Lake Taihu with dense toxic *Microcystis* blooms. *Aquaculture* **2006**, *261*, 1026–1038.
89. Mohamed, Z.A.; Hussein, A.A. Depuration of microcystins in tilapia fish exposed to natural populations of toxic cyanobacteria: A laboratory study. *Ecotoxicol. Environ. Saf.* **2006**, *63*, 424–429.
90. Sipiä, V.; Sjövall, O.; Valtonen, T.; Barnaby, D.L.; Codd, G.A.; Metcalf, J.S.; Kilpi, M.; Mustonen, O.; Meriluoto, J.A.O. Analysis of nodularin-r in eider (*Somateria mollissima*), roach (*Rutilus rutilus*) and flounder (*Platichthus flesus*) liver and muscle samples from the Western Gulf of Finland, northern Baltic Sea. *Environ. Toxicol. Chem.* **2006**, *25*, 2834–2840.
91. Adamovský, O.; Kopp, R.; Hilscherová, K.; Babica, P.; Palíková, M.; Pasková, V.; Navrátil, S.; Marsálek, B.; Bláha, L. Microcystin kinetics (bioaccumulation and elimination) and biochemical responses in common carp (*Cyprinus carpio*) and silver carp (*Hypophthalmichthys molitrix*) exposed to toxic cyanobacterial blooms. *Environ. Toxicol. Chem.* **2007**, *26*, 2687–2693.
92. Osswald, T; Rellán, S.; Carvalho, A.P.; Gago, A., Vasconcelos, V. Acute effects of an anatoxin—a producing cyanobacterium on juvenile fish—*Cyprinus carpio* L. *Toxicon* **2007**, *49*, 693–698.
93. Sipiä, V.; Kankaanpää, H.; Peltonen, H.; Vinni, M.; Meriluoto, J. Transfer of nodularin to three-spined stickleback (*Gasterosteus aculeatus* L.), herring (*Clupea harengus* L.), and salmon (*Salmo salar* L.) in the northern Baltic Sea. *Ecotoxicol. Environ. Saf.* **2007**, *66*, 421–425.
94. Zhang, H.J.; Zhang, J.Y.; Hong, Y.; Chen, Y.X. Evaluation of organ distribuition of microcystins in the freshwater phytoplanktivorous fish *Hypophytalmincthtys molitrix*. *J. Zheijang Univ. Sci. B* **2007**, *8*, 116–120.
95. Wilson, A.E.; Gossiaux, D.C.; Höök, T.O.; Berry, J.P.; Landrum, P.F.; Dyble, J.; Guildford, S.J. Evaluation of the human health threat associated with the hepatotoxin microcystin in the muscle and liver tissues of yellow perch (*Perca flavescens*). *Can. J. Fish. Aquat. Sci.* **2008**, *65*, 1487–1497.
96. Deblois, C.P.; Aranda-Rodriguez, R.; Giani, A.; Bird, D.F. Microcystin accumulation in liver and muscle of tilapia in two large Brazilian hydroelectric reservoirs. *Toxicon* **2008**, *51*, 435–448.

97. Chen, J.; Zhang, D.; Xie, P.; Wang, Q.; Ma, Z. Simultaneous determination of microcystin contaminations in various vertebrates (fish, turtle, duck and water bird) from a large eutrophic Chinese lake, Lake Taihu, with toxic *Microcystis* blooms. *Sci. Total Environ.* **2009**, *407*, 3317–3322.
98. Persson, K.-P.; Legrand, C.; Olsson, T. Detection of nodularin in European flounder (Platichthys flesus) in the west coast of Sweden: Evidence of nodularin mediated oxidative stress. *Harmful Algae* **2009**, *8*, 832–838.
99. Vuorinen, P.J.; Sipiä, V.O.; Karlsson, K.; Keinänen, M.; Furey, A.; Allis, O.; James, K.; Perttilä, U.; Rimaila-Pärnänen, E.; Meriluoto, J.A.O. Accumulation and effects of nodularin from a single and repeated oral doses of cyanobacterium *Nodularia spumigena* on flounder (*Platichthys flesus* L.). *Arch. Environ. Contam. Toxicol.* **2009**, *57*, 164–173.
100. Zhang, D.; Xie, P.; Liu, Y.; Qiu, T. Transfer, distribution and bioaccumulation of microcystins in the aquatic food web in Lake Taihu, China, with potential risks to human health. *Sci. Total Environ.* **2009**, *407*, 2191–2199.
101. Papadimitriou, T.; Kagalou, I.; Bacopoulos, V.; Leonardos, I.D. Accumulation of microcystins in water and fish tissues: an estimation of risks associated with microcystins in most of the Greek lakes. *Environ. Toxicol.* **2010**, *25*, 418–427.
102. Amé, M.V.; Galanti, L.N.; Menone, M.L.; Gerpe, M.S.; Moreno, V.J.; Wunderlin, D.A. Microcystin-LR, -RR, -YR and -LA in water samples and fishes from a shallow lake in Argentina. *Harmful Algae* **2010**, *9*, 66–73.
103. Clemente, Z.; Busato, R.H.; Ribeiro, C.A.O.; Cestari, M.M.; Ramsdorf, W.A.; Magalhães, V.F.; Wosiack, A.C.; Assis, H.C.S. Analyses of paralytic shellfish toxins and biomarkers in a southern Brazilian reservoir. *Toxicon* **2010**, *55*, 396–406.
104. El Ghazali, I.; Saqrane, S.; Carvalho, A.P.; Ouahid, Y.; Del Campo, F.F.; Vasconcelos, V.; Oudra, B. Effects of the microcystin profile of a cyanobacterial bloom on growth and toxin accumulation in common carp *Cyprinus carpio* larvae. *J. Fish Biol.* **2010**, *76*, 1415–1430.
105. El Ghazali, I.; Saqrane, S.; Carvalho, A.P.; Ouahid, Y.; Del Campo, F.F.; Oudra, B.; Vasconcelos, V. Effect of different microcystin profiles on toxin bioaccumulation in common carp (*Cyprinus carpio*) larvae via *Artemia* nauplii. *Ecotoxicol. Environ. Saf.* **2010**, *73*, 762–770.
106. Nyakairu, G.W.A.; Nagawa, C.B.; Mbabazi, J. Assessment of cyanobacteria toxins in freshwater fish: A case study of Murchison Bay (Lake Victoria) and Lake Mburo, Uganda. *Toxicon* **2010**, *55*, 939–946.
107. Berry, J.; Lee, E.; Walton, K.; Wilson, A.; Bernal-Brooks, F. Bioaccumulation of microcystins associated with a persistent cyanobacterial bloom in Lago de Patzcuaro (Michoacan, México). *Environ. Toxicol. Chem.* **2011**, *30*, 1621–1628.
108. White, S.H.; Duivenvoorden, L.J.; Fabbro, L.D.; Eaglesham, G.K. Mortality and toxin bioaccumulation in *Bufo marinus* following exposure to *Cylindrospermopsis raciborskii* cell extracts and live cultures. *Environ. Pollut.* **2007**, *147*, 158–167.
109. Nasri, H.; El Herryb, S.; Bouaïcha, N. First reported case of turtle deaths during a toxic *Microcystis* spp. bloom in Lake Oubeira, Algeria. *Ecotoxicol. Environ. Saf.* **2008**, *71*, 535–544.

110. Krienitz, L.; Ballot, A.; Kotut, K.; Wiegand, C.; Pütz, S.; Metcalf, J.S.; Codd, G.A.; Pflugmacher, S. Contribution of hot spring cyanobacteria to the mysterious deaths of Lesser Flamingos at Lake Bogoria, Kenya. *FEMS Microbiol. Ecol.* **2003**, *43*, 141–148.
111. Sipiä, V.O.; Karlsson, K.A.; Meriluoto, J.A.O.; Kankaanpää, H.T. Eiders (*Somateria mollissima*) obtain nodularin, a cyanobacterial hepatotoxin, in Baltic Sea food web. *Environ. Toxicol. Chem.* **2003**, *23*, 1256–1260.
112. Pašková, V.; Adamovský, O.; Pikula, J.; Skočovská, B.; Band'ouchová, H.; Horáková, J.; Babica, P.; Maršálek, B.; Hilscherová, K. Detoxification and oxidative stress responses along with microcystins accumulation in Japanese quail exposed to cyanobacterial biomass. *Sci. Total Environ.* **2008**, *398*, 34–47.

© 2011 by the authors; licensee MDPI, Basel, Switzerland. This article is an open access article distributed under the terms and conditions of the Creative Commons Attribution license (http://creativecommons.org/licenses/by/3.0/).
